# Systemic delivery of glycosylated-PEG-masked oncolytic virus enhances targeting of antitumor immuno-virotherapy and modulates T and NK cell infiltration

**DOI:** 10.7150/thno.87498

**Published:** 2023-10-02

**Authors:** Yuzhi Liang, Bing Wang, Qingjing Chen, Xingyue Fu, Chenwei Jiang, Zhiwen Lin, Qiuyu Zhuang, Yongyi Zeng, Xiaolong Liu, Da Zhang

**Affiliations:** 1 The United Innovation of Mengchao Hepatobiliary Technology Key Laboratory of Fujian Province, Mengchao Hepatobiliary Hospital of Fujian Medical University, Fuzhou 350025, P. R. China.; 2 CAS Key Laboratory of Design and Assembly of Functional Nanostructures, Fujian Institute of Research on the Structure of Matter, Chinese Academy of Sciences, Fuzhou 350002, P. R. China.; 3 Mengchao Med-X Center, Fuzhou University, Fuzhou 350116, P. R. China.; 4 Liver Disease Center, The First Affiliated Hospital of Fujian Medical University, Fuzhou 350005, P. R. China.; 5 Fujian Agriculture and Forestry University, Fuzhou 350002, P. R. China.

**Keywords:** Oncolytic Virus, ASGPR, galactose-PEG polymer chain, systemic delivery, immuno-virotherapy.

## Abstract

**Rationale:** Immuno-virotherapy has emerged as a promising approach for cancer treatment, as it directly and cytotoxically eliminates tumors with systemic immune stimulation. However, the clinical efficacy of this approach remains limited by inappropriate delivery routes, robust antiviral responses, and the tumor immunosuppressive microenvironment.

**Methods:** To address these challenges, we propose a surface engineering strategy that masks oncolytic herpes simplex virus (oHSV) with a galactose-polyethylene-glycol (PEG) polymer chain to minimize host antiviral responses and selectively targets tumors by limiting exposure to circulation upon systemic administration. We evaluated the antitumor efficacy of glycosylated-PEG-oHSV by examining tumor growth in animal models and analyzing tumor-infiltrating CD8^+^T cells and NK cells in the tumor microenvironment (TME). To assess the neutralizing antibody levels after systemic administration of glycosylated-PEG-oHSV, we utilized a mouse model and measured oHSV-specific IgG.

**Results:** We demonstrate that the glycosylated-PEG modified oHSV does not affect the replication of oHSV yet exhibits high specificity to the asialoglycoprotein receptor (ASGPR) overexpressed in hepatocellular carcinoma cells. This results in selectively targeting cancer cells and deep penetration into tumors while avoiding spreading into the brain. Our approach also effectively reduces oHSV-specific neutralizing antibody levels to mitigate host antiviral immune response. Notably, our glycosylated-PEG-oHSV alleviates the immunosuppressive microenvironment within tumors by reducing regulatory T cells, augmenting the infiltration of activated CD8^+^T cells and NK cells with increasing release of anti-tumor cytokines, to impede tumor progression.

**Conclusion:** Our findings offer a widely applicable and universal strategy to enhance cancer immuno-virotherapy through systemic administration of non-genetically engineered oncolytic viruses. This approach has the potential to overcome the limitations of current immune-virotherapy strategies and may improve clinical outcomes for cancer patients.

## Introduction

Cancer immunotherapy is a promising treatment strategy that has revolutionized oncology 1-3, with immune checkpoint inhibitors (ICIs) being a universal approach for the treatment of advanced malignancies such as advanced hepatocellular carcinoma (HCC) 4-10. Despite the wide use of ICI therapy, the clinical benefit remains limited, with an objective response rate (ORR) of only 10-20% in patients 11-13. This is partially attributed to the "cold" tumor state, characterized by the limited presence of cytotoxic T-cell lymphocytes (CTLs). Additional factors include variable patient immune responses and immunosuppression within the tumor microenvironment (TME) 14-18, which is always the presence of large amounts of regulatory T cells (Tregs) in tumors. Tregs are CD4^+^T cells that suppress the antitumor activities of CTLs, thereby promoting tumor progression 19. Therefore, there is a strong need to develop new therapeutic strategies to enrich CTLs in tumors and further block Treg negative feedback to enhance immunotherapy.

Oncolytic virus (OV) therapy is a powerful tool in converting "cold" tumors unresponsive to immunotherapy into "hot" tumors through cytocidal effects while sparing normal cells 20, 21. The OV-mediated release of tumor-associated antigens (TAAs) and damage-associated molecular patterns (DAMPs) can serve as an *in-situ* tumor vaccine, to activate immune cells, recruit immune effector cells, and produce systemic immunologic effects to inhibit tumor growth 22-26. However, local administration of OVs for solid tumors can be challenging due to the compactness and high pressure of the TME, which poses a significant hurdle for clinical translation 21, 27. As a result, systemic administration of OVs is preferred to broaden their clinical applications. However, this approach remains extremely challenging due to the pre-existing neutralizing antibodies in the host, which can induce a strong antiviral immune response that leads to severe side effects and rapid virus clearance 28. Ultimately, this has led to unsatisfactory anti-tumor immune responses in clinical settings, hindering the desired therapeutic effects 29-31.

To address these issues, researchers have developed various systemic delivery strategies for OVs, including genetically engineered, serum albumin-mediated, stem cell/cell membrane-mediated, and liposome-mediated oncolytic adenovirus, to specifically target and accumulate the virus in tumors and reduce the anti-adenovirus neutralizing antibodies. These approaches offer promise in enhancing the efficacy of immuno-virotherapy for cancer 32-35. Compared to genetic engineering or cell/cell membrane modification strategies, polymer-based adenovirus modification strategies offer unique features, such as low cost, convenience, and flexibility 30. Recent work has focused on polymer-based engineering strategies for oncolytic vaccinia viruses that utilize electrostatic or hydrophobic interactions 36, but challenges regarding stability during systemic administration remain widespread.

**Scheme 1:**
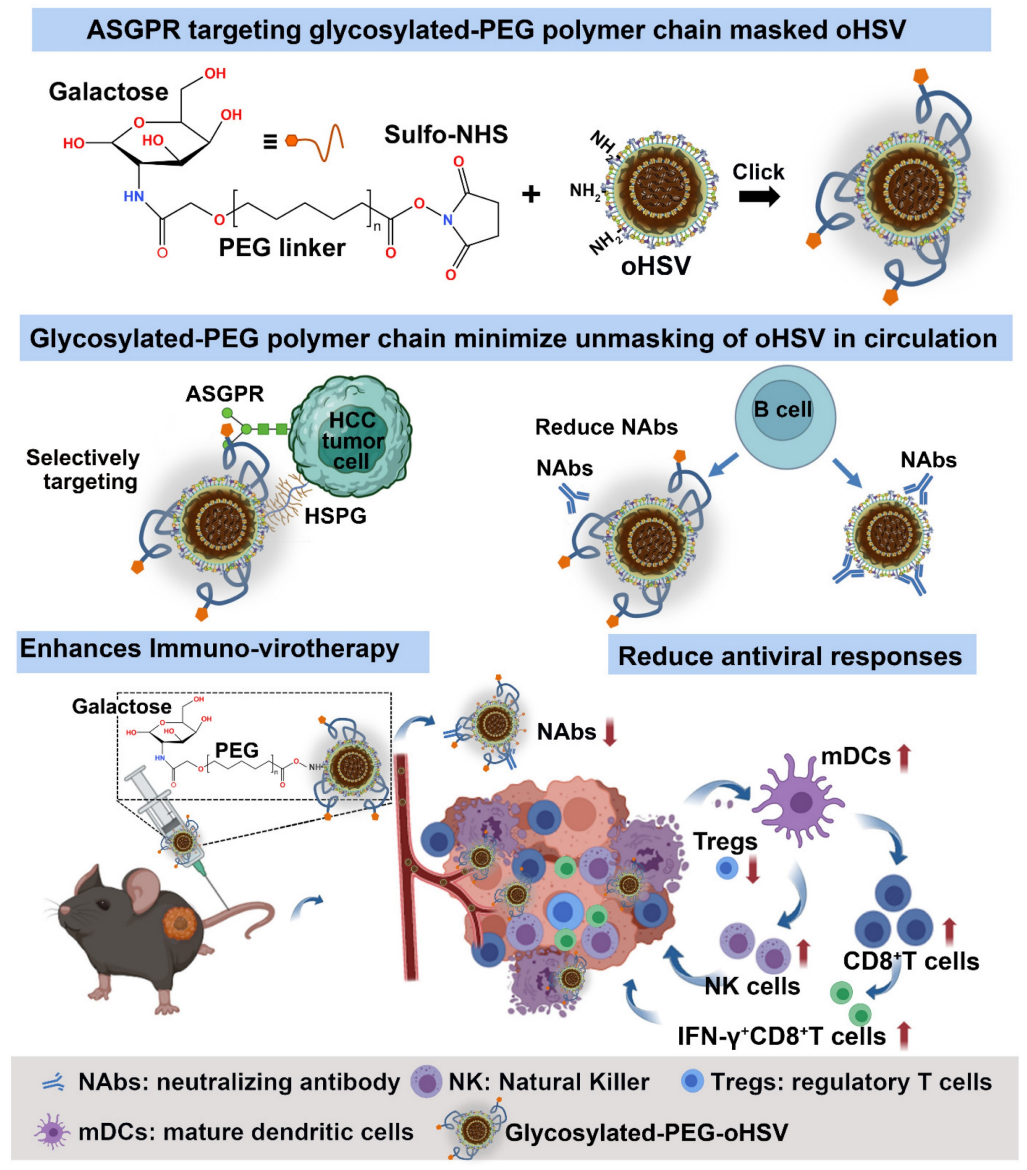
Schematic illustration of the preparation and tumor targeting principle of the galactose-polyethylene glycol polymer chain modified oHSV (glycosylated-PEG-oHSV), and the modulation of the tumor microenvironment by glycosylated-PEG-oHSV to enhance antitumor immunity. The resulting glycosylated-PEG-oHSV exhibited targeted delivery to tumors and increased cell infection, while also reducing infection of healthy cells. Upon systemic administration, the glycosylated-PEG-oHSV effectively and specifically destroyed HCC tumors, stimulated the immune responses, and reshaped the immunosuppressive tumor microenvironment by decreasing Tregs and increasing infiltration of IFN-γ^+^CD8^+^T cells, CD8^+^T cells, and NK cells within tumors, which led to efficient inhibition of HCC tumor growth.

In this study, we used a covalent coupling approach to develop a galactose-poly (ethylene glycol) polymer (glycosylated-PEG)-armed oncolytic herpes simplex virus (glycosylated-PEG-oHSV) for more effective cancer immuno-virotherapy in mouse models of HCC through systemic administration (**Scheme 1**). This optimized glycosylated-PEG-oHSV selectively infected and killed cancer cells by targeting the asialoglycoprotein receptor (ASGPR) present in HCC cells 37, while efficiently reducing oHSV-neutralizing antibody levels and decreasing the number of regulatory T-cells within the TME after systemic administration. As a result, it demonstrated potent cytocidal activity against Hepa1-6 tumors in mice and increased the release of anti-tumor cytokines levels, which lead to higher CD8^+^T cell and NK cell infiltration into tumors, therefore effectively preventing tumor growth.

## Results

### The impact of glycosylated-PEG on cell infection and oncolytic activity of oHSV

**Figure 1:**
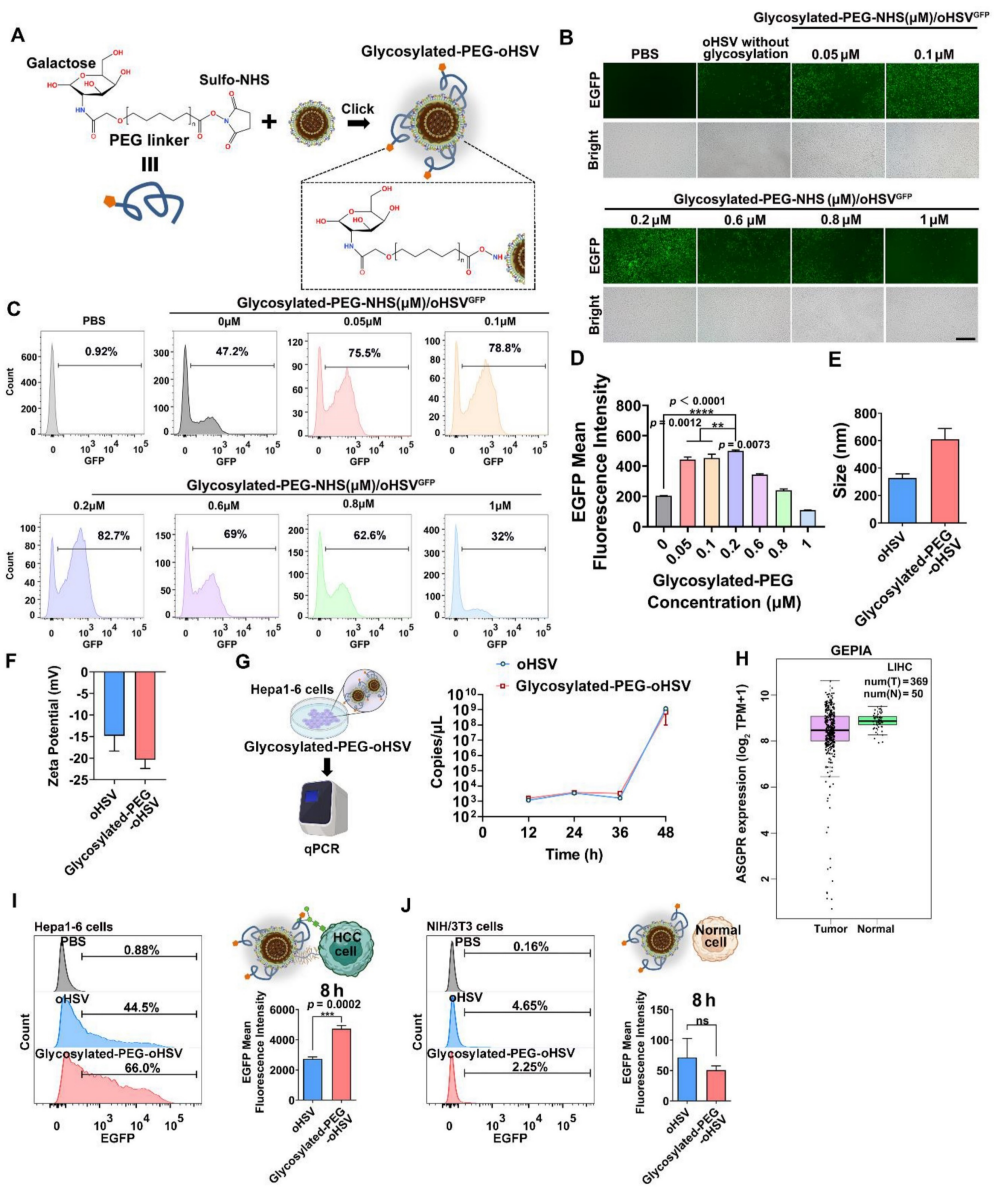
**(A)** Schematic illustration of the preparation of glycosylated-PEG conjugation on oHSV. **(B)** Bright-field and green fluorescence images of infected Hepa1-6 cells, with varying degrees of glycosylation on the oHSV. The photo was imaged 24 h after co-incubation. **(C)** Flow cytometry analysis and **(D)** quantification of EGFP expression were performed on Hepa1-6 cells after treatment with either oHSV or oHSV conjugated with varying concentrations of glycosylated-PEG for 24 h. The nano size **(E)** and zeta potential **(F)** of oHSV and glycosylated-PEG-oHSV. **(G)** The virus replication of oHSV before and after modification with glycosylated-PEG in 293T cells qualified by qPCR. **(H)** ASGPR expression in HCC and normal tissues was determined by TCGA HCC transcriptome sequencing. **(I)** and **(J)** Flow cytometry was performed on Hepa1-6 and NIH/3T3 cells treated with either oHSV or glycosylated-PEG-oHSV. Statistical analysis was performed with t-test or ANOVA analysis, *p<0.05, ****p<0.0001, (n = 3). Data are presented as mean ± SD.

To optimize the conjugation of glycosylated-PEG to oHSV, we used different concentrations of glycosylated-PEG (ranging from 0.05 μM to 1 μM) to covalently attach it to lysine on the envelope of oHSV (at a concentration of 1 MOI) using the lysine-amide coupling strategy for 24 h** (Figure 1A)**. The resulting glycosylated-PEG-oHSV was then co-cultured with a mouse liver tumor cell line (Hepa1-6) at different concentrations of glycosylated-PEG and then observed using fluorescence microscopy. Our observations showed that the cell infection efficiency of glycosylated-PEG-oHSV, indicated by the GFP (green) fluorescence in Hepa1-6 cells, was concentration-dependently increased in the range of 0 to 0.2 μM of conjugated glycosylated-PEG at the constant virus titer (**Figure 1B**). To further quantify the infection efficiency of glycosylated-PEG-oHSV, flow cytometry (FCM) was used. **As shown in Figure 1C-D, and Figure S1,** the highest infection efficiency of glycosylated-PEG-oHSV was observed at 0.2 μM conjugated glycosylated-PEG in Hepa1-6 cells, which was significantly higher than any other concentration of tested glycosylated-PEG. To confirm the successful modification of glycosylated-PEG on the oHSV, we measured the size distribution and zeta potential of glycosylated-PEG-oHSV. **As shown in Figure 1E-F**, the hydrodynamic diameter of glycosylated-PEG-oHSV increased from 326.93 ± 25.29 nm to 610.57 ± 63.48 nm, suggesting some aggregation of oHSV likely due to the unique topological structure of glycoproteins on the surface of oHSV, and the zeta potential of glycosylated-PEG-oHSV changed from -14.8 ± 2.91 to -20.37 ± 1.64, respectively. To confirm the actual size of oHSV with or without glycosylated-PEG modification, the oHSV was further characterized by a transmission electron microscope (TEM). The average size of oHSV was approximately 227.6 nm, and the glycosylated-PEG-oHSV was approximately 247.2 nm (**Figure S2**). Additionally, we determined the conjugated glycosylated-PEG from oHSV using a colorimetric fluorometric method provided by the manufacturer** (Figure S3)**. The results showed that the glycosylated-PEG-oHSV contained 6.60 ± 0.51 ng glycosylated-PEG/μL /1 × 10^8^ pfu. To investigate whether the replication of oHSV was affected by the conjugation of glycosylated-PEG, we measured the replication of both the unmodified oHSV and the optimized glycosylated-PEG-oHSV in Hepa1-6 cells.

We then quantified the copies of viral genomes of oHSV using quantitative polymerase chain reaction (qPCR) and found that the optimized glycosylated-PEG-oHSV did not affect the replication of oHSV at any of the time points measured (12 to 48 h). Specifically, we observed that the viral genome copies for the optimized glycosylated-PEG-oHSV were similar to those of unmodified oHSV **(Figure 1G)**. These results suggest that the optimized glycosylated-PEG-oHSV has a high cell infection capacity in Hepa1-6 cells without compromising its replication.

**Figure 2:**
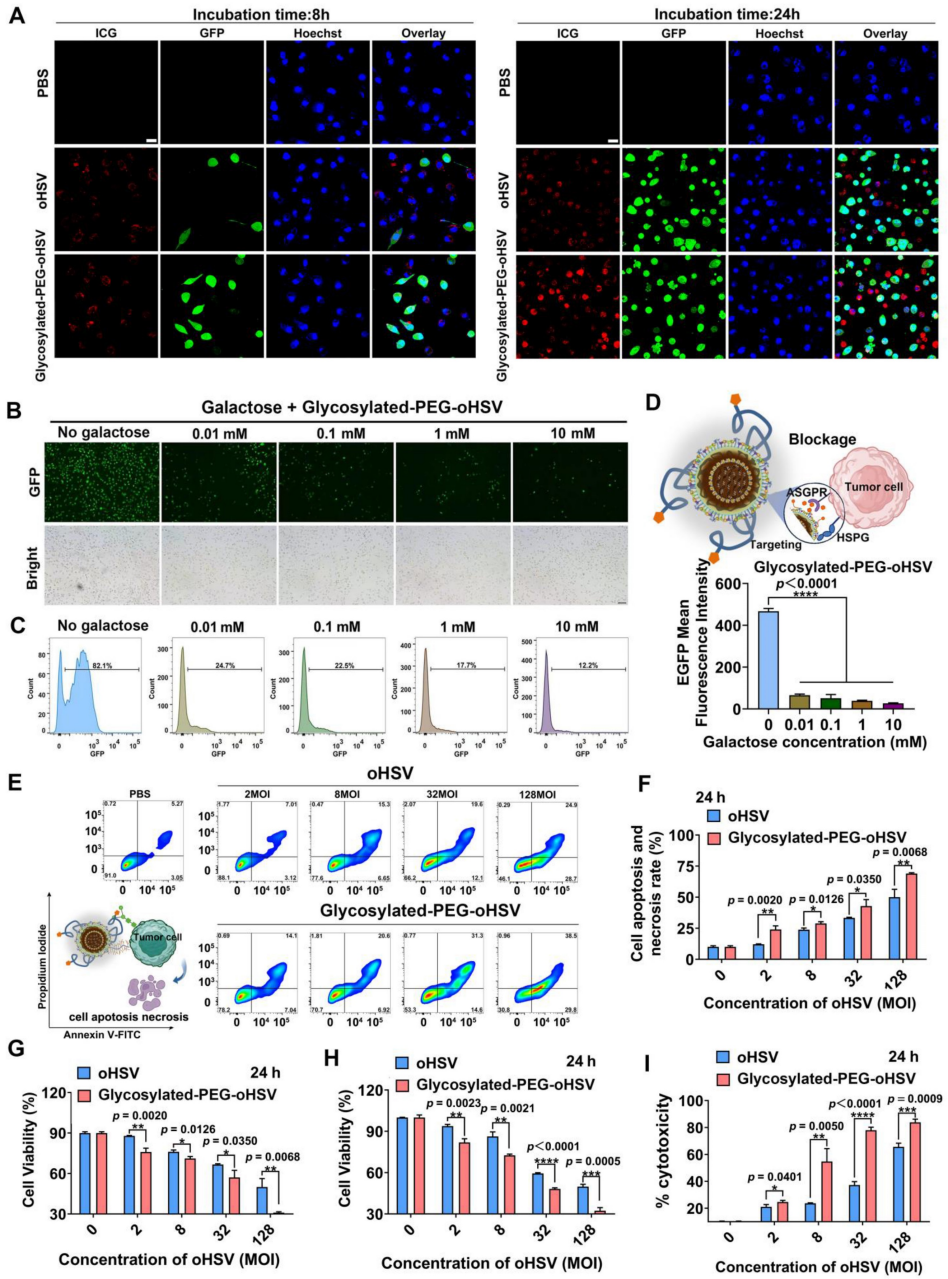
**(A)** CLSM images of Hepa1-6 cells treated with ^ICG^glycosylated-PEG-oHSV or ^ICG^ oHSV after 8 h and 24 h, respectively. The red color represents ICG, the green color represents EGFP expression, and the blue color represents Hoechst (nuclear). Scale bar, 20 μm. **(B)** Cell infection efficiency of glycosylated-PEG-oHSV or oHSV in Hepa1-6 cells after blocking ASGPR with different galactose concentrations for 1 h through fluorescence imaging and **(C)** flow cytometry analysis. **(D)** Quantification of the EGFP expression by flow cytometry in Hepa1-6 cells after blocking ASGPR with different concentrations of galactose and then treated with glycosylated-PEG-oHSV. **(E)** and **(F)** Cytolytic activity was determined by co-incubating glycosylated-PEG-oHSV or oHSV with Hepa1-6 cells for 24 h, followed by analysis using FACS with Annexin V-FITC and PI staining. **(G)** The percentage of apoptosis and necrosis in Hepa1-6 cells after indicated treatments (n = 3). Cell viability of Hepa1-6 cells was analyzed by CCK8 assay **(H)** and LDH assay **(I)** after treatment with different titers (0-128 MOI) of oHSV or glycosylated-PEG-oHSV (n = 3). Statistical analysis was performed using ANOVA analysis, **p<0.01, ***p<0.001, ****p<0.0001. Data are presented as mean ± SD.

ASGPR is known to have a high affinity for binding to glycoproteins with exposed terminal galactose or N-acetylgalactosamine residues, which are targets for hepatocellular carcinoma (HCC) therapy, due to its selective expression on liver cancer cells 38, 39. Based on TCGA data, it showed that ASGPR is overexpressed in HCC compared to normal tissues **(Figure 1H).** This makes the use of glycosylated-PEG-oHSV a highly promising potential therapeutic agent for HCC, with an expected enhancement in efficacy and specificity. To investigate the selective targeting ability of glycosylated-PEG-oHSV to the overexpressed ASGPR in Hepa1-6 cells 40-42, we performed co-incubation experiments using glycosylated-PEG-oHSV or oHSV with Hepa1-6 cells for 8 h. Additionally, a control was introduced using NIH/3T3 cells, a fibroblast cell line derived from a mouse NIH/Swiss embryo that has low or no expression of ASGPR. To evaluate the effects of viral infection, we performed a comparative analysis of glycosylated-PEG-oHSV and oHSV-treated Hepa1-6 cells using FCM to measure EGFP expression. Our analysis revealed a higher infection efficiency of the glycosylated-PEG-oHSV treated cells, as evidenced by an EGFP-positive percentage of 62.57 %. In contrast, oHSV-treated Hepa1-6 cells showed an EGFP-positive percentage of only 47 % **(Figure 1I)**. These results were consistent with the glycosylated-PEG-oHSV-treated HepG2 cells (human liver cancer cell line) (**Figure S4**). These data clearly demonstrate the superior targeting ability of glycosylated-PEG-oHSV to Hepa1-6 and HepG2 cells overexpressing ASGPR. Despite the improved infection efficiency of glycosylated-PEG-oHSV in Hepa1-6 cells, the infection of NIH/3T3 cells was minimal. Glycosylated-PEG-oHSV-treated NIH/3T3 cells showed an infection efficiency of only 1.72 ± 0.40%, which was similar to the oHSV-treated NIH/3T3 cells (4.6%) after 8 h of co-incubation **(Figure 1J and Figure S5)**. The weak replication of oHSV in NIH/3T3 cells is mainly attributed to the deletion of certain essential replication genes of the virus. However, after 12 h of co-incubation, the infection efficiency of NIH/3T3 cells treated with oHSV increased to 38.27 %, whereas the infection efficiency of cells treated with glycosylated-PEG-oHSV only reached 19.87 %. This lower efficiency in the glycosylated-PEG-oHSV group in NIH/3T3 cells is likely due to the effect of the PEG polymer chain, suggesting that the glycosylated-PEG masked oHSV has lower toxicity toward NIH/3T3 cells **(Figure S6)**. Confocal imaging (CLSM) of ICG-NHS-labeled glycosylated-PEG-oHSV (^ICG^glycosylated-PEG-oHSV)-treated Hepa1-6 cells was also performed. The results showed a strong red (ICG) fluorescence and green fluorescence (EGFP expression) compared to cells treated with oHSV alone, both at 8 and 24 h after treatment **(Figure 2A)**. These findings suggest that the glycosylated-PEG modification of oHSV efficiently enhances its cellular uptake, which in turn improves its infection efficiency.

To validate the importance of the glycosylated-PEG modification in enhancing cellular uptake of oHSV, we performed blocking experiments using different concentrations of galactose (ranging from 0.10 to 10 mM) to inhibit the ASGPR of Hepa1-6 cells for 1 h prior to co-incubation with glycosylated-PEG-oHSV** (Figure 2B-D)**. Our CLSM imaging and FCM analyses showed that exposure to galactose at concentrations as low as 0.01 mM resulted in a significant reduction of EGFP expression in glycosylated-PEG-oHSV-treated Hepa1-6 cells. The cell-infectivity of glycosylated-PEG-oHSV followed a concentration-dependent inhibitory effect of galactose. These results confirm that the incorporation of glycosylated-PEG into oHSV significantly enhances its effective cellular uptake in Hepa1-6 cells overexpressing ASGPR.

### Glycosylated-PEG enhances the cytolytic activity of oHSV in HCC cells

To evaluate the cytotoxic effect of glycosylated-PEG-oHSV on Hepa1-6 cells, we stained the infected cells with Annexin-V-FITC and Propidium Iodide (PI) after treatment with different viral titers of glycosylated-PEG-oHSV (ranging from 2 to 128 MOI) for a 24 h co-incubation **(Figure 2E-F and Figure S7)**. Our results showed that the percentages of apoptosis and necrosis in tumor cells treated with glycosylated-PEG-oHSV (24.07 % at 2 MOI and 65.7 % at 128 MOI) were significantly higher than the oHSV-treated cells (12.1 % at 2 MOI and 50.03 % at 128 MOI) under the same condition **(Figure 2G).** These results demonstrate the high cytolytic activity of glycosylated-PEG-oHSV compared to non-glycosylated-PEG-oHSV, which can be further verified by the Cell Counting Kit 8 (CCK8) assay by measuring the killing efficiency **(Figure 2H)**.

To further verify the cytolytic effect of glycosylated-PEG-oHSV, we measured the levels of lactate dehydrogenase (LDH) released from the Hepa1-6 cells after treatment with glycosylated-PEG-oHSV or oHSV, respectively** (Figure 2I).** The results showed a significant increase in cell mortality rate, with glycosylated-PEG-oHSV-treated cells reaching 54.74 % as compared to oHSV-treated cells with a mortality rate of 23.66 % (at 8 MOI). Furthermore, the cell mortality rate further increased to 83.84 % in glycosylated-PEG-oHSV treated cells compared to oHSV treated cells with a mortality rate of 65.65 % (at 128 MOI). These results suggest that the incorporation of glycosylated-PEG significantly enhances the oncolytic efficacy of oHSV, resulting in a reduction of Hepa1-6 cell viability.

### Glycosylated-PEG enhances oHSV accumulation and reduces its distribution in normal tissues

**Figure 3:**
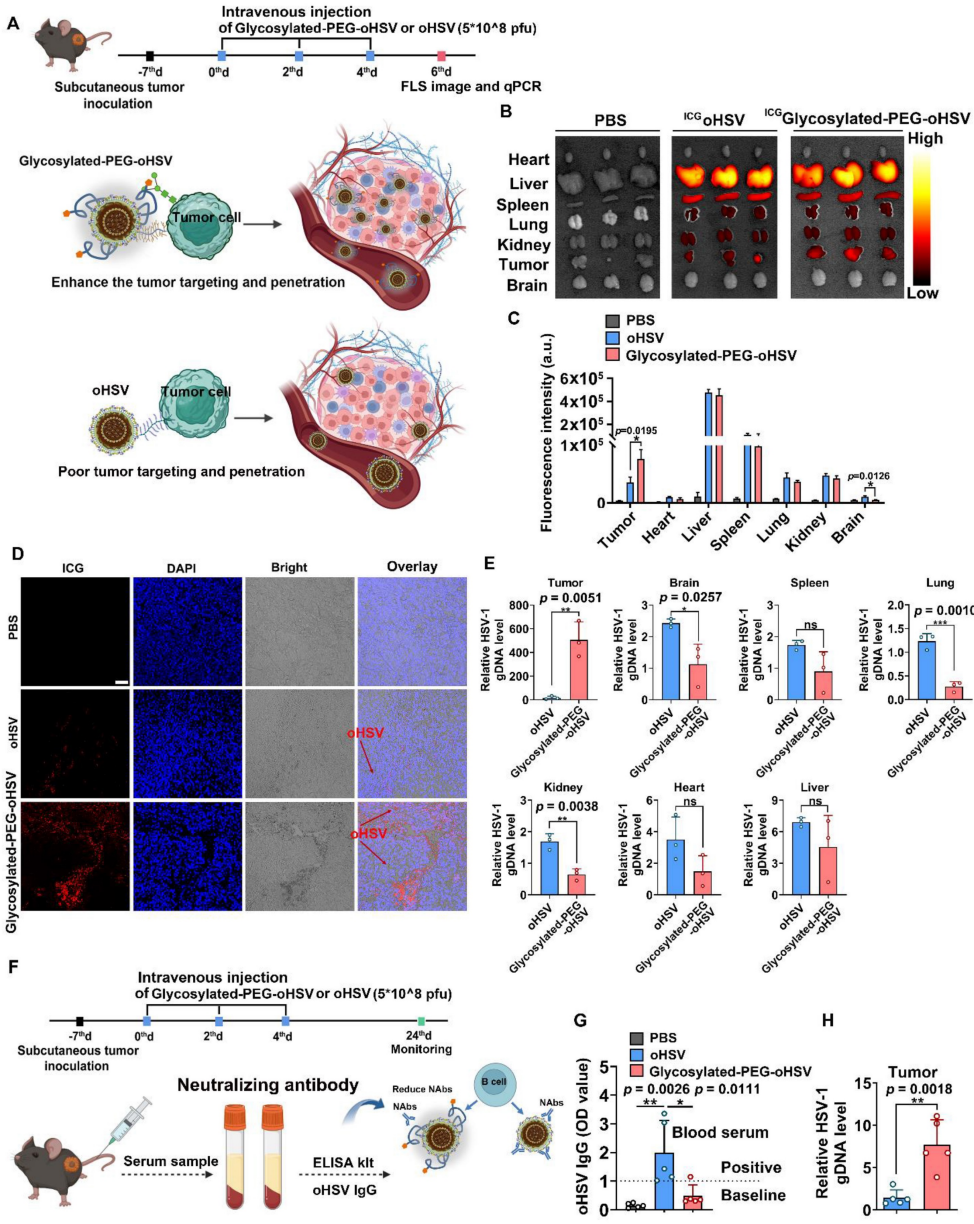
** (A)** Schematic illustration of the process of intravenous injection of glycosylated-PEG-oHSV into C57bL/6 mice three times every two days after subcutaneous inoculation of Hepa1-6 tumor cells to evaluate the antitumor effect of glycosylated-PEG-oHSV. **(B)** and **(C)** *In vivo* fluorescence images and intensity of C57bL/6 mice treated with PBS, ^ICG^oHSV, or ^ICG^glycosylated-PEG-oHSV for 48h of injection. **(D)** CLSM image and distribution of ^ICG^ oHSV or ^ICG^glycosylated-PEG-oHSV in tumor tissues after treatments in C57bL/6 mice as indicated, respectively. Scale bar, 50 μM. **(E)** oHSV genomic DNA levels were measured by RT-qPCR after the indicated treatments in the indicated organs, represented by oHSV gD DNA levels (n = 3; data are shown as means ± SD). **(F)** and **(G)** The process of intravenous injection of glycosylated-PEG-oHSV or oHSV three times, and then collection of serum for oHSV IgG analysis and quantification by ELISA kit, (n = 5). **(H)** oHSV genomic DNA levels in tumors were measured by RT-qPCR after intravenous injection of oHSV or glycosylated-PEG-oHSV (n = 5; data are shown as means ± SD). Statistical analysis was performed using ANOVA analysis, *p<0.05, **p<0.01, ***p<0.001, ****p<0.0001. Data are expressed as mean ± SD.

Based on the enhanced cell infection and cytotoxicity observed with glycosylated-PEG-oHSV, we investigated its tumor targeting ability and killing efficacy in *in*-*vivo* mouse tumor models (**Figure 3A**). Male C57bL/6 mice (4-5 weeks old) were subcutaneously inoculated with Hepa1-6 cells. When the established tumor volume reached approximately 100 mm^3^, the mice were injected intravenously (*i.v.*) with ^ICG^glycosylated-PEG-oHSV or ICG-NHS labeled oHSV (^ICG^oHSV) three times every two days** (Figure 3A)**. The treated mice were later sacrificed, and their tumors, heart, liver, spleen, lung, kidney, and brain were isolated and imaged using the *In Vivo* Fluorescence Imaging System. As shown in** Figure 3B-C**, systemic administration of ^ICG^glycosylated-PEG-oHSV resulted in higher fluorescence intensity within tumors compared to administration of ^ICG^oHSV. We further validated the efficient accumulation of ^ICG^glycosylated-PEG-oHSV within tumors by sectioning and imaging the tumors using CLSM** (Figure 3D)**. The images showed a significant accumulation of^ ICG^glycosylated-PEG-oHSV with red fluorescence in both the peripheral and central areas of the tumors. In contrast, ^ICG^oHSV showed weak red signals only at the tumor margin. These findings provide evidence for the successful accumulation and penetration of glycosylated-PEG-oHSV into tumors after modification with glycosylated-PEG. Interestingly, the mice treated with ^ICG^glycosylated-PEG-oHSV exhibited lower brain intensity than those treated with ^ICG^oHSV, suggesting that the glycosylated-PEG polymer chains acted as a protective barrier, referred to as "mask" to reduce the infection in healthy tissues. This protective barrier helped to minimize the spread of oHSV to vital organs, particularly in the brain. oHSV infections, even mild ones, can lead to neuronal damage similar to that observed in neurodegenerative diseases such as Alzheimer's disease 43.

To further confirm these findings, we performed quantitative polymerase chain reaction (qPCR) to detect the oHSV genomic DNA (gDNA) in tumors, heart, liver, spleen, lung, kidney, and brain. As shown in **Figure 3E**, the gDNA levels in tumors treated with glycosylated-PEG-oHSV were significantly higher than those in tumor treated with oHSV. However, gDNA levels in the brain, lung, and kidney of mice treated with glycosylated-PEG-oHSV were much lower than those of oHSV. We also evaluated the blood biochemistry and routine indexes and found them to be within the normal range after the injection of glycosylated-PEG-oHSV at 24 days post-injection **(Figure S8)**. Moreover, the systemic administration of oncolytic viruses (OVs) has always been plagued by a strong antiviral reaction with neutralizing antibodies within the host, resulting in severe toxicity and unsatisfactory anti-tumor immune responses 29-31. In addition, we analyzed the levels of oHSV-specific antibodies of glycosylated-PEG-oHSV with the use of an ELISA kit. The mice were treated with glycosylated-PEG-oHSV or oHSV by systemic administration, with a frequency of three times every two days. Serum was collected from the mice on day 24 after injection **(Figure 3F)**. The results demonstrated that mice treated with oHSV alone had significantly higher levels of oHSV-specific IgG than those treated with glycosylated-PEG-oHSV **(Figure 3G)**. Additionally, we examined the genomic DNA (gDNA) of oHSV in tumors *via* qPCR on day 24. Our results showed that the gDNA levels in tumors treated with glycosylated-PEG-oHSV were significantly higher than those treated with oHSV alone (**Figure 3H**). This finding suggests that glycosylated-PEG effectively reduces the host antiviral reaction towards oHSV and efficiently enhances the accumulation of oHSV in tumors.

### Glycosylated-PEG-oHSV enhances the antitumor therapeutic efficacy

**Figure 4:**
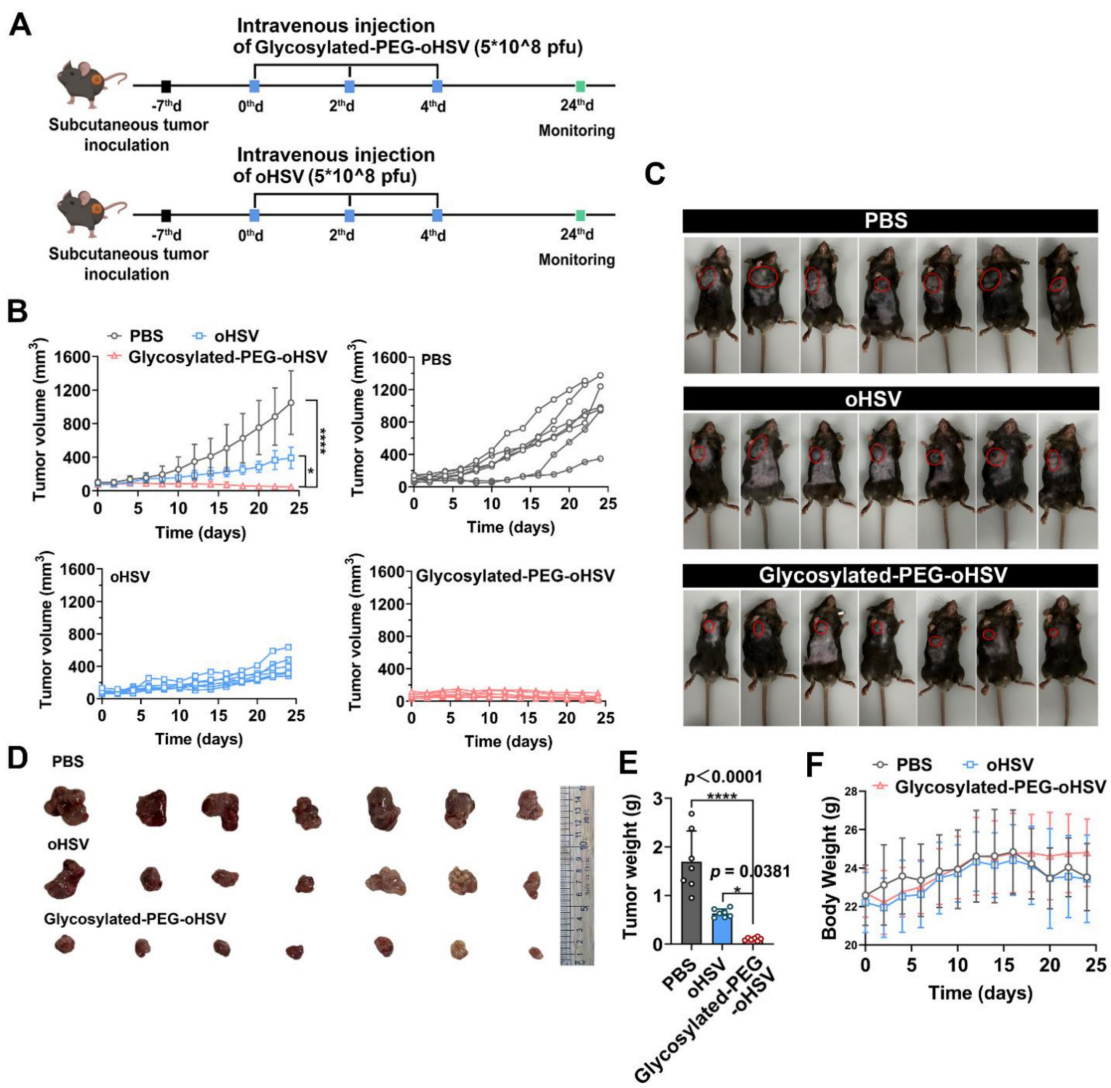
** (A)** Schematic illustration of the process of intravenous injection of glycosylated-PEG-oHSV into C57bL/6 mice and its subsequent antitumor effect. **(B)** Tumor volumes of mice treated with PBS, oHSV, or glycosylated-PEG-oHSV, respectively. (n = 7 mice per group; data are shown as means ± SD). **(C)** Photomicrograph of mice and **(D)*** ex*-tumor after indicated treatments (n = 7). **(E)** The *ex*-tumor weights of mice on day 24 after treatment with PBS, oHSV, or glycosylated-PEG-oHSV, respectively, (n = 7). **(F)** The body weights of mice after treatments as indicated, (n = 7). Statistical analysis was performed using ANOVA analysis, *p<0.05, **p<0.01, ***p<0.001, ****p<0.0001. Data are expressed as mean ± SD, (n = 7).

Next, we evaluated the *in vivo* cytolytic activity of glycosylated-PEG-oHSV by *i.v.* administration of glycosylated-PEG-oHSV with either oHSV or PBS as the control groups **(Figure 4A)**. The results showed that although *i.v.* injection of oHSV alone delayed tumor growth, but it failed to completely inhibit tumor progression, probably due to the strong antiviral immune response that resulted in an unsatisfactory therapeutic effect. However, *i.v.* injection of glycosylated-PEG-oHSV completely inhibited tumor progression in all mice (7/7), whereas the non-glycosylated-PEG modified oHSV and PBS control groups showed no inhibition of tumor progression (0/7 and 0/7, respectively) **(Figure 4B)**. The photographs of the *ex*-tumor and tumor weight further confirmed these results **(Figure 4C-E)**, indicating the excellent cytolytic activity and antitumor efficacy of glycosylated-PEG-oHSV. Moreover, glycosylated-PEG-oHSV exhibited remarkable therapeutic efficacy without causing any significant adverse effects, such as body weight loss, in the treated mice. This is in contrast to the oHSV-treated group and the PBS group, where mice showed progressive weight loss over time **(Figure 4F)**. These findings distinguish the use of glycosylated-PEG-oHSV from oHSV alone and suggest that glycosylated-PEG modification may potentially improve the safety profile of oHSV-based therapies.

**Figure 5:**
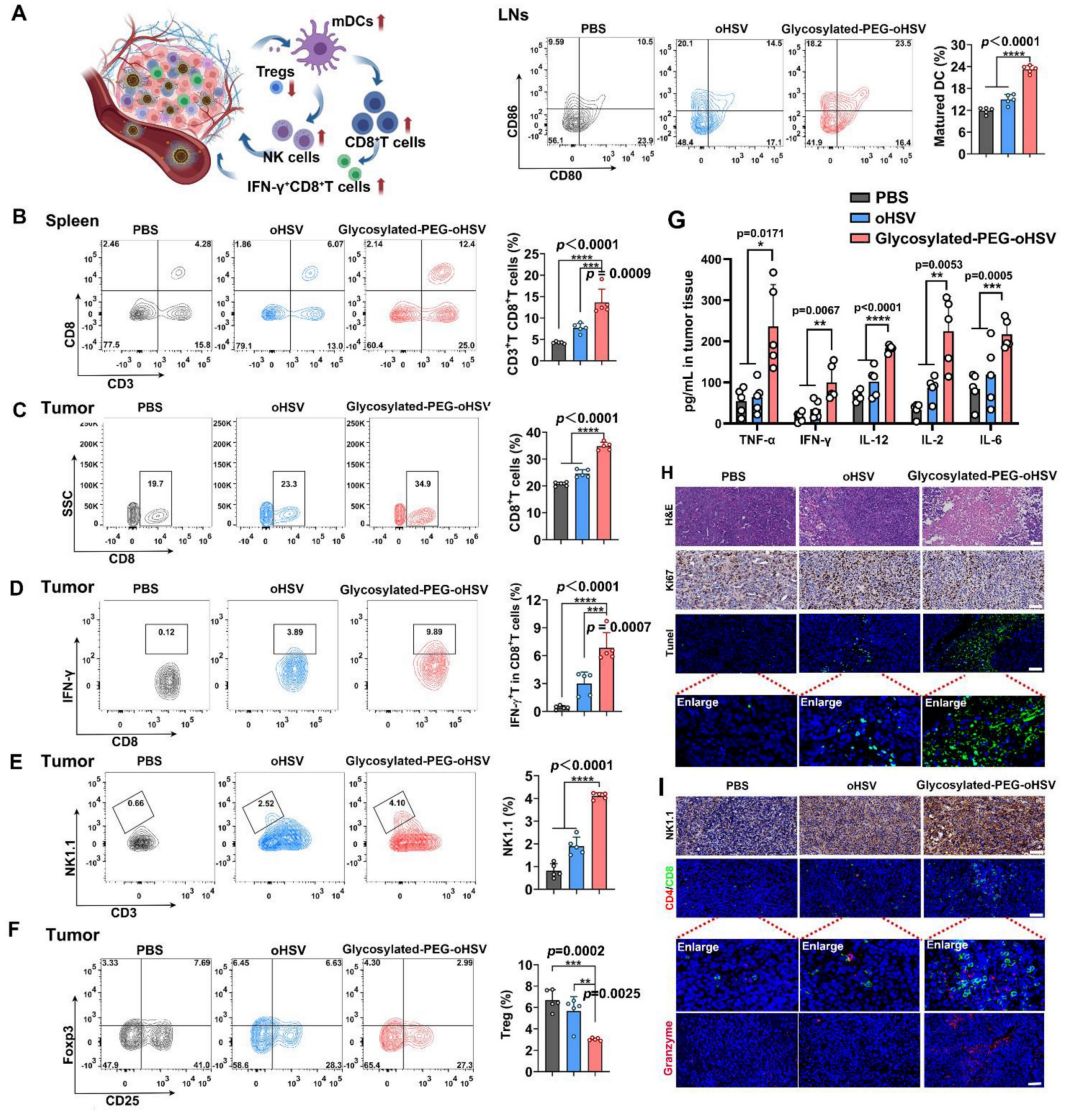
** (A)** The percentage of CD80 and CD86 co-expression in CD11c^+^BMDCs isolated from LNs of mice after treatment with PBS, oHSV, or glycosylated-PEG-oHSV, (n = 5). **(B)** The percentage of CD3^+^CD8^+^T cells in the spleen and **(C)** tumor tissues after treatment with PBS, oHSV, or glycosylated-PEG-oHSV, respectively (n = 5). **(D)** The percentage of activated IFN-γ^+^CD8^+^T cells in tumor tissues after treatment with PBS, oHSV, or glycosylated-PEG-oHSV, respectively (n = 5). **(E)** The percentage of NK1.1^+^CD3^+^NK cells in tumors after treatment with PBS, oHSV, or glycosylated-PEG-oHSV, respectively (n = 5). **(F)** The percentage of Foxp3^+^CD25^+^T cells in tumors after treatment with PBS, oHSV, or glycosylated-PEG-oHSV, respectively (n = 5). Statistical analysis was performed by ANOVA analysis, *p<0.05, **p<0.01, ***p<0.001, ****p<0.0001. Data are expressed as mean ± SD. **(G)** The levels of the cytokines TNF-α, IFN-γ, IL-12, IL-2, and IL-6 were measured in tumors isolated from the differently treated mice using ELISA analysis, (n = 5). **(H)** H&E images of tumors after treatment with PBS, oHSV, or glycosylated-PEG-oHSV. Immunohistochemical analysis of Ki67 staining of tumor sections at day 24. The CLSM image of TUNEL staining of tumor sections and tumor-infiltrating lymphocytes (TILs) on day 24. **(I)** Immunofluorescence analysis of NK1.1 antibodies of NK cells in tumors, or CD4^+^T cell (red), CD8^+^T cell (green), and granzyme (pink) staining of tumor sections after indicated treatments. The nucleus was stained by DAPI (blue). Scale bar, 50 μm.

Furthermore, we assessed the immunologic impact of glycosylated-PEG-oHSV by collecting tumor-draining lymph nodes (LNs) after treatment. Upregulation of surface co-stimulatory molecules such as CD80 and CD86 on mature lymphoid DCs is important for activating T cell responses and promoting their differentiation into cytotoxic T cells 44. Therefore, we employed FCM analysis to measure the expression of co-stimulatory receptors (CD80^+^CD86^+^) in CD11c^+^DCs** (Figure 5A and Figure S9).** The results showed that approximately 23.24 % of the lymphoid DCs in the glycosylated-PEG-oHSV-treated groups were in a maturation state compared to the PBS-treated (11.88 %) or oHSV-treated (14.96 %) groups. These results indicated that glycosylated-PEG-oHSV was effective in inducing tumor oncolysis, which in turn induced the maturation of lymphoid DCs. To further investigate whether the oncolysis of tumors induced by glycosylated-PEG-oHSV could enhance the response of CD8^+^T cells, we examined the population of CD8^+^CD3^+^T cells in the spleens of mice that received various treatments **(Figure 5B and Figure S10)**. The results showed a significant increase in CD3^+^CD8^+^T cells (13.66 %) in mice treated with glycosylated-PEG-oHSV compared to the PBS-treated group (4.26 % of CD3^+^CD8^+^T cells) or oHSV-treated group (7.70 % of CD3^+^CD8^+^T cells). Besides, we also analyzed the proportion of CD3^+^CD4^+^T cells in tumors and found that the tumor bearing mice treated with glycosylated-PEG-oHSV contained a relatively higher percentage of CD3^+^CD4^+^T cells compared to tumors treated with PBS or oHSV alone (**Figure S11**). These results suggest that the glycosylated-PEG-oHSV induced tumor oncolysis effectively enhances the responses of CD3^+^CD8^+^T cells and CD3^+^CD4^+^ helper T cells.

In addition, we evaluated tumor-infiltrating IFN-γ^+^CD8^+^T cells since IFN-γ^+^CD8^+^ T cells play a critical role in cell-mediated immunity 45. We observed a significant increase in the percentage of CD3^+^CD8^+^T cells (34.8 %) and activated IFN-γ^+^CD8^+^T cells (6.85%) in mice treated glycosylated-PEG-oHSV compared to the PBS-treated group (20.64 % of CD3^+^CD8^+^T cells and 0.42 % of IFN-γ^+^ CD8^+^T cells) or oHSV treated group (24.6 % of CD3^+^CD8^+^T cells and 3 % of IFN-γ^+^CD8^+^T cells) **(Figure 5C-D and Figure S12-13)**. These findings provide evidence that glycosylated-PEG-oHSV effectively suppressed tumor growth by enhancing the infiltration of activated CD8^+^T cells into tumors. In addition, oncolytic viruses can induce necroptotic cell death, which leads to a significant increase in natural killer (NK) cell activation, further improving anti-tumor efficacy 46. To investigate the effects of glycosylated-PEG-oHSV on NK cells, we assessed the infiltration of NK cells in tumors using FACS analysis. Our results showed a significant increase in the infiltration of activated CD3^-^NK1.1^+^NK cells (4.11 %) in tumors from mice treated with glycosylated-PEG-oHSV, compared to those treated with PBS (0.82 %) or oHSV (1.90 %) **(Figure 5E and Figure S14)**. These results provide evidence that glycosylated-PEG-oHSV induces NK cell activation and enhances anti-tumor efficacy.

Foxp3^+^CD25^+^CD4^+^ regulatory T (Treg) cells can suppress the function of cytotoxic T lymphocytes (CTLs), which are crucial for tumor cell recognition and killing 47. Therefore, we next evaluated the population of Tregs in tumors and found that glycosylated-PEG-oHSV was more effective in reducing the number of Tregs in tumors (3.02 %) compared to both PBS-treated (6.69 %) and oHSV-treated (5.66 %) groups **(Figure 5F and Figure S15).** These results are consistent with a previously reported phenomenon observed during oHSV treatment, suggesting that the reduction of Tregs may be a promising strategy to enhance the immune-virotherapy of oHSV 48. Furthermore, we measured the cytokines in tumors by ELISA. We found that glycosylated-PEG-oHSV treatment efficiently increased the levels of TNF-α, IFNγ, IL12, IL-2, and IL-6 compared to oHSV treatment alone **(Figure 5G)**. These analyses demonstrated that glycosylated-PEG-oHSV efficiently reduced Tregs in tumor tissues and enhanced the infiltration of effector T cells and NK cells to induce a potent anti-tumor immune response, resulting in suppressed tumor growth. Furthermore, the observed therapeutic efficacy was further confirmed through immunohistochemical analysis and immunofluorescence staining of Ki67, H&E, and TUNEL **(Figure 5H and Figure S16)**. In addition, staining with NK1.1, CD8, CD4 antibodies and granzyme further confirmed the efficient infiltration of NK cells and T cells into tumors after glycosylated-PEG-oHSV treatment (**Figure 5I**). Importantly, the flexibility and small size of glycosylated-PEG make it a promising strategy for modifying viruses to target specific tumors of interest by altering the target ligand or viruses. These findings strongly support the development of glycosylated-PEG polymer chain-armed viruses for tumor immuno-virotherapy in the future.

## Discussion

The use of oncolytic viruses (OVs) as *in-situ* cancer vaccines is a promising approach as they can induce immunogenic cell death of cancer cells and release tumor-associated antigens (TAAs) and damage-associated molecular patterns (DAMPs), thereby promoting an anti-tumor immune response. However, the concurrent induction of a strong antiviral immune response can hinder anti-tumor immunity by leading to rapid viral clearance 21, 28, 49. This issue is particularly challenging for OVs derived from oHSV, as pre-existing neutralizing antibodies in patients can inhibit viral replication 50. In addition, multiple OV injections may generate neutralizing antibodies, limiting the therapeutic efficacy of OVs. Therefore, reducing the activation of the antiviral immune response is crucial for the successful application of OVs in clinical therapy. In this study, we used the galactose-polyethylene glycol polymer to covalently conjugate to the envelope of oHSV, which served as a "Mask" to significantly reduce cross-reactive antibodies in mouse immunity and was confirmed by oHSV specific-IgG* via* an ELISA kit. This outcome is similar to other documented strategies to reduce antiviral immunity, such as using coatings of cell-derived vesicle, polyethyleneimine, lipid or graphene to shield OVs from immune components 51-53. However, these protective coatings come with several challenges such as storage and stability issues, high manufacturing costs, potential toxicity, and scalability difficulties. Our modification strategy provides a simple and convenient covalent coupling approach to retarget oHSV to tumor cells.

Clinical development of OVs remains challenging as most require intra-tumoral administration, which is often difficult for solid tumors because of their compactness and high pressure, potentially leading to post-injection bleeding 54. Emerging preclinical evidence has shown that intravenous administration of OVs offers advantages over intra-tumoral injection, such as the ability to expose all lesions and eliminate the need for complex localization devices. Upon systemic administration, the prepared glycosylated-PEG-oHSV demonstrated highly specific targeting to ASGPR overexpressing tumor cells while sparing the brain, lung, and kidney, which was confirmed by using NIR-II fluorescence imaging and quantified viral replication by qPCR. This approach offers a potential improvement over previous strategies for intravenous delivery of OVs (Enadenotucirev), as these OVs still face challenges related to strong neutralization factors that limit the effective dose of available virus resulting in lower response rates compared to intra-tumoral delivery 55.

Furthermore, extensive evidence has documented the presence of Treg cells following oHSV infection and anti-oHSV vaccination 56. Studies have shown that Tregs, a unique subpopulation of CD4^+^T cells, hinder the antitumor effects of CTLs generated by oHSV-induced immunogenic cell death (ICD) of tumor cells, thereby leading to tumor progression 57, 58. Nevertheless, our study yielded promising results, as the systemic administration of glycosylated-PEG-oHSV significantly decreased the number of Tregs present in the tumor microenvironment, thereby reversing the immunosuppressive state induced by Tregs. In particular, enhancing the potential of oncolytic oHSV to activate CD8^+^T cells is crucial for immuno-virotherapy. Additionally, oHSV can induce necroptotic cell death, which leads to a significant increase in NK cell activation and further improves anti-tumor efficacy 59. In this study, we found that the glycosylated-PEG-oHSV also induced a higher infiltration of IFN-γ^+^CD8^+^T cells and NK cells into tumors, promoting a healthier anti-tumor response and effective tumor growth prevention. The glycosylated-PEG-oHSV achieved excellent cytocidal activity against tumors in mouse models of HCC, suggesting that the non-genetically modified glycosylated-PEG-oHSV holds great promise for future targeted immuno-virotherapy applications. Despite the promising results, it still needs to be tested in clinical trials before it can be adapted for widespread clinical use.

In summary, we have developed a glycosylated-PEG-oHSV and successfully delivered the virus to targeted tumor sites with deep penetration into tumors by systemic administration, resulting in efficient prevention of tumor progression. The glycosylated-PEG-oHSV exhibited remarkable features, including tumor-specific targeting, enhanced cell infection ability, reduced brain infection with minimized side effects, flexibility and small size, reduced host antiviral response and Tregs in the tumor microenvironment, and increased infiltration of IFN-γ^+^CD8^+^T cells and NK cells into tumors after systemic administration. In addition, glycosylated-PEG-oHSV was shown to significantly inhibit tumor growth by reshaping the immunosuppressive tumor microenvironment. Based on these exciting findings, we believe that glycosylated-PEG polymer chain-armed viruses represent a promising strategy for HCC immuno-virotherapy with the potential for clinical translation.

## Methods

### Reagents

Galactose-polyethylene glycol polymer chain (glycosylated-PEG, 35kDa) was purchased from Tianjin Jenkem Technology Co. Ltd (Tianjin, China). Annexin V-FITC/PI and CCK8 kit were purchased from Dojindo Laboratories. Anti-CD11c-APC, anti-CD4-FITC, anti-CD86-PE-Cy7, anti-CD3-APC, anti-Foxp3-PE-Cy7, anti-IFN-γ-PE-Cy7, anti-CD80-PE, anti-CD8-PE, anti-3-FITC, anti-NK1.1-APC, and anti-CD25-Percp-Cy5.5 were purchased from BioLegend, Inc (San Diego, CA, USA). LDH kits were purchased from Abcam (Cambridge, UK).

### Cell Culture and animals

Hepa1-6 cells (isolated from the BW7756 tumor that arose spontaneously in the C57L/J mouse strain) and NIH/3T3 cells were cultured in DMEM (supplemented with 100 IU/mL penicillin-streptomycin and 10% fetal bovine serum) at 37^o^C with 5% CO_2_. C57BL/6 mice (male, 4-5 weeks old, SPF) were purchased from China Wushi, Inc (Shanghai, China). The animals were cared for according to the guidelines outlined in the "Guide for the Care and Use of Laboratory Animals of Mengchao Hepatobiliary Hospital of Fujian Medical University", and all animal procedures were approved by the Animal Ethics Committee of Mengchao Hepatobiliary Hospital of Fujian Medical University.

### Virus propagation and titration

The oncolytic virus GFP-HSV-1, which was created by the insertion of the GFP sequence into G47delta BAC, was used. The Vero cell line was used for virus propagation, and the viral plaque assay was performed to determine the titers of the amplified virus as described previously. To quantify the levels of HSV-1 genomic DNA, samples from infected tissue or cell cultures were extracted using the TIANamp Genomic DNA Kit (TIANGEN, Germany). Subsequently, qPCR was performed using specific primers for HSV-1 genomic DNA:

Forward primer for HSV-1 gD: 5'-acgactggacggagattaca-3'

Reverse primer for HSV-1 gD: 5'-ggagggcgtacttacaggag-3'.

### Synthesis of Glycosylated-PEG-oHSV

To obtain glycosylated-PEG-oHSV, 0.007 g of glycosylated-PEG was dissolved in 1 mL of ddH_2_O to achieve a concentration of 200 μM. The resulting solution was then mixed with oHSV (DMEM medium) at a volume ratio of 1:1, and the mixture was shaken on an oscillator (600 rpm) at 4°C in a refrigerator for 24 h. The glycosylated-PEG with sulfo -NHS can react with the lysine-amide of oHSV, to form an amido bond (-CO-NH-) on the envelope of oHSV. Finally, the glycosylated-PEG-oHSV was purified by high-speed centrifugation within a DMEM medium, and then stored in DMEM at 4 ^o^C for further usage.

### Optimization of the binding ratio between glycosylated-PEG and oHSV

To determine the optimal ratio of glycosylated-PEG binding to oHSV, various concentrations of glycosylated-PEG (0, 0.05, 0.1, 0.2, 0.6, 0.8 or 1 μM) were mixed with oHSV (1 MOI), incubated at 4 °C in a refrigerator for 24 h, and then purified by high-speed centrifugation to obtain glycosylated-PEG-oHSV. Afterward, Hepa1-6 cells were seeded in 24-well plates at a density of 1 × 10^5^ cells per well. After 12 h incubation, the supernatant was removed, and a mixture of 1.2 × 10^5^ pfu glycosylated-PEG-oHSV and 1 mL of medium was added to each well of the experimental group, while a mixture of 1.2 × 10^5^ pfu oHSV and 1 mL of medium was added to the control group. The cells were incubated at 37^o^C with 5% CO_2_ for 24 h. Afterward, the treated cells were imaged using a fluorescence microscope and analyzed by flow cytometry (FACS), respectively.

### Detection of the copy number changes before and after oHSV modification

To evaluate the copy number changes before and after oHSV modification, we designed the primers 5'-CTGTGCTATCCCCATCACGG-3' and 5'-GTTCTGGCTGCGTGGCGTTG-3' for PCR amplification. After gel electrophoresis, the DNA was recovered and the concentration was measured. The copy number was calculated using the formula: copy number / μl = DNA concentration × 10^-9^ × 6.02 × 10^23^ / (fragment length × 660), and the standards were obtained after dilution. The oHSV, glycosylated-PEG-oHSV, and Hepa1-6 cells were incubated for 12 h, 24 h, 36 h, and 48 h, and the DNA from each group was extracted for qPCR analysis. The ct value was determined from the standard curve using the ct value and copy number of the standard: y = -1.439ln(x) + 34.594, R^2^ = 0.996. The copy number can be obtained.

### Determination of galactose content before and after oHSV modification

To quantify the content of glycosylated-PEG modification of oHSV, we used the galactose kit for the assay. 1 mM (1 nmol / μL) standard solution was prepared by diluting 10 μL of 100 mM (100 nmol / μL) galactose standard solution with 990 μL of galactose assay buffer. After thorough mixing, 0, 2, 4, 6, 8, and 10 μL of the 1 mM galactose standard solution were added to a 96-well plate to prepare standard solutions of 0 (assay blank), 2, 4, 6, 8 and 10 nM / well. Galactose assay buffer was added to each well to reach a final volume of 50 mL, liquid samples can be assayed directly. The addition of Galactose Assay Buffer brought the final volume of the sample to 50 μL. The reaction premix of 50 μL was added to each well, and the mixture was thoroughly mixed using a horizontal shaker or pipetting. The reaction was incubated for 60 min at 37 °C, protected from light. The absorbance at 570 nm (A570) was then measured.

### Tumor cell-targeting and cellular uptake of Glycosylated-PEG-oHSV

To evaluate the targeting ability of glycosylated-PEG-oHSV on tumor cells, Hepa1-6 cells were used as the experimental group, while NIH/3T3 cells were utilized as the control. Both cell lines were seeded in 24-well plates at a density of 1 × 10^5^ per well and allowed to incubate overnight. Subsequently, 1.2 × 10^5^ pfu of glycosylated-PEG-oHSV mixed with 1 mL of medium was added to each well of the experimental group, while 1.2 × 10^5^ pfu of oHSV mixed with 1 mL of medium was added to the control group. The plates were then incubated at 37°C with 5% CO_2_ for 8 and 12 h, and the cells were collected for fluorescence intensity analysis using FACS.

To evaluate the cellular uptake of glycosylated-PEG-oHSV in Hepa1-6 cells, ICG-NHS (50 µg) was used to label glycosylated-PEG-oHSV or oHSV. ^ICG^glycosylated-PEG-oHSV and ^ICG^oHSV were then obtained through centrifugation and resuspension of the precipitate. Hepa1-6 cells (2 × 10^5^ per well) were seeded onto 35 mm glass-bottom Petri dishes, and then treated with 3 × 10^6^ pfu ^ICG^glycosylated-PEG-oHSV or ^ICG^oHSV. The cells were incubated at 37 °C with 5% CO_2_ for 8h and 24 h and subsequently washed with PBS buffer (1 ×, pH 7.4). Confocal microscopy (CLSM) (LSM 780, Germany) was used to image the treated cells, with cell nuclei stained with Hoechst 33342 (excitation at 405 nm), ICG-NHS dye excited at 633 nm, and EGFP excited at 488 nm.

### Blocking ASGPR receptor upon glycosylated-PEG-oHSV cell-infection

To investigate the impact of galactose on the recognition of ASGPR by glycosylated-PEG-oHSV, Hepa1-6 cells were seeded in 6-well plates at a density of 3 × 10^5^ cells per well and incubated overnight. Different concentrations of galactose (0.01 mM, 0.1 mM, 1 mM, 10 mM) were added to the cells and incubated for 1 h. The supernatant was then removed, and glycosylated-PEG-oHSV was added to the cells. The plates were incubated at 37°C with 5% CO_2_ for 24 h. The effect of virus infection was evaluated using fluorescence microscopy and FACS to analyze the fluorescence intensity.

### Targeted oncolytic effect of glycosylated-PEG-oHSV

To evaluate the lysis-killing effect of glycosylated-PEG-oHSV on tumor cells, Hepa1-6 cells were seeded at a density of 1 × 10^5^ cells per well in a 24-well plate with varying concentrations of oHSV and glycosylated-PEG-oHSV (2, 8, 32, 128 MOI), and incubated in 37°C with 5% CO_2_ for 24 h. All cells were collected completely by centrifugation and resuspended with 1 × buffer from the Annexin V / PI apoptosis / necrosis kit. Annexin-V and PI dyes (5 μL each) for apoptosis/necrosis staining were added to stain the cells for 15 minutes at room temperature avoiding light exposure before analysis by FACS.

In addition, Hepa1-6 cells were seeded in 96-well plates at a density of 1 × 10^4^ cells per well, and treated with varying concentrations of oHSV or glycosylated-PEG-oHSV (2, 8, 32, 128 MOI) for 24 h. The supernatant was aspirated and washed once with PBS buffer. Then, 10 μL CCK-8 and 90 μL DMEM medium were added to each well and incubated at 37°C with 5% CO_2_ for 1 h, protected from light. The absorption at 450 nm was detected to determine the cell viability by a Spectra 206 Max M5 spectrophotometer according to the previous protocol 18.

Furthermore, we assessed cell mortality using the LDH kit. Hepa1-6 cells were seeded at a density of 1 × 10^5^ cells per well in a 24-well plate and treated with varying concentrations of oHSV and glycosylated-PEG-oHSV (2, 8, 32, 128 MOI) for 24 h at 37°C with 5% CO_2_. The supernatant was then collected, and 120 µL of supernatant of each sample was mixed with 60 µL of LDH assay working solution in a 96-well plate. The mixture was incubated for 30 min at room temperature on a shaker protected from light, and the absorbance was measured at 490 nm to calculate the cell mortality.

The LDH detection assay was performed using the Abcam LDH cytotoxicity assay kit. Supernatant from the cell culture medium was collected and the cytotoxicity rate was determined using the LDH Cytotoxicity Detection Kit. The cytotoxicity rate was calculated as (exp. value - low control) / (high control - low control) × 100%. The low control was supernatant from untreated Hepa1-6 cells. The high control was supernatant from Hepa1-6 cells treated with 1% Triton X-100.

### Bio-distribution of Glycosylated-PEG-oHSV* in vivo*

To evaluate the *in vivo* distribution of glycosylated-PEG-oHSV in mice after systemic administration, C57BL/6 mice were subcutaneously injected with 3 × 10^6^ cells of Hepa1-6 cell suspension to establish tumor xenografts. When the tumor volume reached approximately 100 mm^3^, the established tumor mice were intravenously injected with ^ICG^glycosylated-PEG-oHSV or ^ICG-^oHSV, with PBS injection as the control. After 48 h of injection, the mice were euthanized by cervical dislocation, and major organs, including heart, spleen, kidney, lung, liver, tumor, and brain, were isolated and fluorescently imaged using the IVIS®Spectrum *In Vivo* Imaging System. Additionally, the expression of the oHSV genome in each tissue was determined by qPCR. The distribution of glycosylated-PEG-oHSV or oHSV in tumor sections was further imaged by CLSM, with cell nuclei stained with DAPI (excitation at 405 nm) and ICG-NHS dye excited at 633 nm.

### Detection of oHSV specific antibodies *in vivo*

To detect the production of antiviral serum in mice, oHSV or glycosylated-PEG-oHSV (5 × 10^8^ pfu) was intravenously injected into mice three times per two days. On day 24, blood samples were obtained from the treated mice and stored at -20 ^o^C. An enzyme immunoassay kit was used to detect the presence of mouse oHSV antibody IgG (oHSV IgG) in the serum.

### Antitumor therapy of Glycosylated-PEG-oHSV* in vivo*

To assess the antitumor therapy of glycosylated-PEG-oHSV *in vivo*, the 4-5 weeks male C57BL/6 mice were subcutaneously injected with sterile PBS containing Hepa1-6 cells (3 × 10^6^). The animals were housed using an Individual Ventilated Caging (IVC) System from Shenzhen Suhang Technology Co., Ltd. (Shenzhen, China). When the tumor volume reached about 50 mm^3^, the male C57BL/6 mice were randomly divided into three treatment groups and housed in separate cages per group: PBS, oHSV (5 × 10^8^ pfu), glycosylated-PEG-oHSV (5 × 10^8^ pfu), and intravenous injected for three times every two days. The tumor volume (V) was calculated using the following equation: V = A × B ^ 2 / 2, where A and B are the long and short diameter of the tumor (mm), respectively.

### The antitumor immune response of Glycosylated-PEG-oHSV* in vivo*

To investigate the antitumor immune response of glycosylated-PEG-oHSV *in vivo*, C57BL/6 mice were sacrificed 24 days after treatment. Tumor-draining lymph nodes were collected, and the lymphoid DCs were stained with anti-CD11c-APC (eBioscience, USA), anti-CD80-PE (eBioscience, USA), and anti-CD86-PE-Cy7 (eBioscience, USA) antibodies, and analyzed using FACS. Additionally, the isolated tumors were completely clipped and then digested using collagenase type IV (1 mg/mL), hyaluronidase (0.2 mg/mL), and deoxyribonuclease I (0.02 mg/mL) at 37^o^C for 2 h. The resulting single-cell suspension was then stained with various antibodies for the analysis of infiltrated T cells, regulatory T cells (Tregs), and natural killer (NK) cells. Specifically, CD8^+^T cells were stained with anti-CD3-APC and anti-CD8-PE antibodies; Tregs cells were stained with anti-CD3-APC, anti-CD4-FITC (eBioscience, USA), anti-CD25-PerCP-Cy5.5 (eBioscience, USA), and anti-Foxp3-PE-Cy7 (eBioscience, USA) antibodies; NK cells were stained with anti-CD3-FITC (eBioscience, USA) and anti-NK1.1-APC (eBioscience, USA) antibodies; IFN-γ^+^CD8^+^T cells in the tumor were analyzed by FACS using anti-CD3-APC (eBioscience, USA), anti-CD8-PE (eBioscience, USA), and anti-IFN-γ-PE-Cy7 (eBioscience, USA) antibodies. Furthermore, to analyze the CD3^+^ CD8^+^T cells in the spleen, the spleen from treated mice was isolated. The cells were obtained by syringe rinsing of the spleen and then collected by density gradient centrifugation through FicollPaqueTM PREMIUM sterile solution, incubated with 5% BSA for 15 minutes, and washed once with PBS. Subsequently, the collected cells were stained with anti-CD3-APC and anti-CD8-PE antibodies, and analyzed by FACS.

### Histological evaluation

The tumor-bearing mice were sacrificed on day 24 collected after receiving treatments, and stained with hematoxylin and eosin (H&E), as well as Ki67 (R&D Systems, USA), or TUNEL (R&D Systems, USA), and NK1.1 antibodies (ThermoFisher, USA). The immunofluorescence staining of tumor sections was using CD4 and CD8 antibodies provided by Service (China).

### Statistical analysis

Statistical analysis of all data was performed using one-way analysis of variance (ANOVA) for comparisons among multiple groups. While a two-tailed Student's t-test was used for comparisons between the two groups. GraphPad Prism 8.0 software was used to perform these analyses. The significance level was set at *p < 0.05 for statistical significance. **p < 0.01, ***p < 0.001, ****p < 0.0001. Pearson's correlation coefficient was used to evaluate the correlation matrices. All the data are presented as means ± standard through at least three experiments. Data are expressed as mean ± SD.

## Supplementary Material

Supplementary figures.

## Figures and Tables

**Table 1:** Designed primers for RT-qPCR.

Target	Forward	Reverse
Murine HSV-1 Gapdh	5'-CCACTCACGGCAAATTCAAC-3'	5'-CTCCACGACATACTCAGCAC-3'
Murine HSV-1 gD	5'-ACGACTGGACGGAGATTACA-3'	5'-GGAGGGCGTACTTACAGGAG-3'
Murine HSV-1 standards	5'-CTGTGCTATCCCCATCACGG-3'	5'-GTTCTGGCTGCGTGGCGTTG-3'

## References

[1] Waldman AD, Fritz JM, Lenardo MJ (2020). A guide to cancer immunotherapy: from T cell basic science to clinical practice. Nat Rev Immunol.

[2] Chen H, Li Z, Qiu L, Dong X, Chen G, Shi Y (2022). Personalized neoantigen vaccine combined with PD-1 blockade increases CD8+ tissue-resident memory T-cell infiltration in preclinical hepatocellular carcinoma models. J Immunother Cancer.

[3] Cai Z, Su X, Qiu L, Li Z, Li X, Dong X (2021). Personalized neoantigen vaccine prevents postoperative recurrence in hepatocellular carcinoma patients with vascular invasion. Mol Cancer.

[4] Cheng AL, Hsu C, Chan SL, Choo SP, Kudo M (2020). Challenges of combination therapy with immune checkpoint inhibitors for hepatocellular carcinoma. J Hepatol.

[5] Carlino MS, Larkin J (2021). Long GV. Immune checkpoint inhibitors in melanoma. Lancet.

[6] Oliva M, Spreafico A, Taberna M, Alemany L, Coburn B, Mesia R (2019). Immune biomarkers of response to immune-checkpoint inhibitors in head and neck squamous cell carcinoma. Ann Oncol.

[7] Heeke AL, Tan AR (2021). Checkpoint inhibitor therapy for metastatic triple-negative breast cancer. Cancer Metastasis Rev.

[8] Llovet JM, Castet F, Heikenwalder M, Maini MK (2022). Immunotherapies for hepatocellular carcinoma. Nat Rev Clin Oncol.

[9] Rijnders M, de Wit R, Boormans JL, Lolkema MP, van der Veldt AA (2017). A systematic review of immune checkpoint inhibition in urological cancers. Eur Urol.

[10] Ding L, Zhang X, Yu P, Peng F, Sun Y, Wu Y (2023). Genetically engineered nanovesicles mobilize synergistic antitumor immunity by ADAR1 silence and PDL1 blockade. Mol Ther.

[11] Aykan NF, Özatlı T (2020). Objective response rate assessment in oncology: current situation and future expectations. World J Clin Oncol.

[12] Liu YT, Sun ZJ (2021). Turning cold tumors into hot tumors by improving T-cell infiltration. Theranostics.

[13] Wong JSL, Kwok GGW, Tang V, Li BCW, Leung R, Chiu J (2021). Ipilimumab and nivolumab/pembrolizumab in advanced hepatocellular carcinoma refractory to prior immune checkpoint inhibitors. J Immunother Cancer.

[14] Lu C, Rong D, Zhang B, Zheng W, Wang X, Chen Z (2019). Current perspectives on the immunosuppressive tumor microenvironment in hepatocellular carcinoma: challenges and opportunities. Mol Cancer.

[15] Andersen BM, Faust Akl C (2021). Glial and myeloid heterogeneity in the brain tumour microenvironment. Nat Rev Cancer.

[16] Ullman NA, Burchard PR (2022). Immunologic strategies in pancreatic cancer: making cold tumors hot. J Clin Oncol.

[17] Zhang Y, Zhang Z (2020). The history and advances in cancer immunotherapy: understanding the characteristics of tumor-infiltrating immune cells and their therapeutic implications. Cell Mol Immunol.

[18] Zhang D, Jiang C, Zheng X, Lin Z, Zhuang Q, Xie H (2023). Normalization of tumor vessels by lenvatinib-based metallo-nanodrugs alleviates hypoxia and enhances calreticulin-mediated immune responses in orthotopic HCC and organoids. Small.

[19] Li C, Jiang P, Wei S, Xu X, Wang J (2020). Regulatory T cells in tumor microenvironment: new mechanisms, potential therapeutic strategies and future prospects. Mol Cancer.

[20] Jahan N, Ghouse SM, Martuza RL, Rabkin SD (2021). *In situ* cancer vaccination and immunovirotherapy using oncolytic HSV. Viruses.

[21] Shalhout SZ, Miller DM, Emerick KS, Kaufman HL (2023). Therapy with oncolytic viruses: progress and challenges. Nat Rev Clin Oncol.

[22] Ylösmäki E, Malorzo C, Capasso C, Honkasalo O, Fusciello M, Martins B (2018). Personalized cancer vaccine platform for clinically relevant oncolytic enveloped viruses. Mol Ther.

[23] Macedo N, Miller DM, Haq R, Kaufman HL (2020). Clinical landscape of oncolytic virus research in 2020. J Immunother Cancer.

[24] Ferrucci PF, Pala L, Conforti F, Cocorocchio E (2021). Talimogene Laherparepvec (T-VEC): An intralesional cancer immunotherapy for advanced melanoma. Cancers.

[25] Suryawanshi YR, Zhang T, Razi F, Essani K (2020). Tanapoxvirus: from discovery towards oncolytic immunovirotherapy. J Cancer Res Ther.

[26] Wu M, Li H, Zhang C, Wang Y, Zhang C, Zhang Y (2023). Silk-gel powered adenoviral vector enables robust genome editing of PD-L1 to augment immunotherapy across multiple tumor models. Adv Sci.

[27] Ma R, Li Z, Chiocca EA, Caligiuri MA, Yu J (2023). The emerging field of oncolytic virus-based cancer immunotherapy. Trends Cancer.

[28] Krysko DV, Garg AD, Kaczmarek A, Krysko O, Agostinis P, Vandenabeele P (2012). Immunogenic cell death and DAMPs in cancer therapy. Nat Rev Cancer.

[29] Chaurasiya S, Fong Y, Warner SG (2020). Optimizing oncolytic viral design to enhance antitumor efficacy: progress and challenges. Cancers.

[30] Todo T (2012). Active immunotherapy: oncolytic virus therapy using HSV-1. Adv Exp Med Biol.

[31] Ban W, Guan J, Huang H, He Z, Sun M, Liu F (2022). Emerging systemic delivery strategies of oncolytic viruses: a key step toward cancer immunotherapy. Nano Res.

[32] Liu Y, Ye T, Maynard J, Akbulut H, Deisseroth A (2006). Engineering conditionally replication- competent adenoviral vectors carrying the cytosine deaminase gene increases the infectivity and therapeutic effect for breast cancer gene therapy. Cancer Gene Ther.

[33] Lv P, Liu X, Chen X, Liu C, Zhang Y, Chu C (2019). Genetically engineered cell membrane nanovesicles for oncolytic adenovirus delivery: a versatile platform for cancer virotherapy. Nano Lett.

[34] Harrington KJ, Puzanov I, Hecht JR, Hodi FS, Szabo Z, Murugappan S (2015). Clinical development of talimogene laherparepvec (T-VEC): a modified herpes simplex virus type-1-derived oncolytic immunotherapy. Expert Rev Anticancer Ther.

[35] Du W, Seah I, Bougazzoul O, Choi G, Meeth K (2017). Stem cell-released oncolytic herpes simplex virus has therapeutic efficacy in brain metastatic melanomas. Proc Natl Acad Sci.

[36] Garofalo M, Bellato F, Magliocca S, Malfanti A, Kuryk L (2021). Polymer coated oncolytic adenovirus to selectively target hepatocellular carcinoma cells. Pharmaceutics.

[37] Kim Y, Jo M, Schmidt J, Luo X, Prakash TP, Zhou T (2019). Enhanced potency of GalNAc-conjugated antisense oligonucleotides in hepatocellular cancer models. Mol Ther.

[38] D'Souza AA, Devarajan P V (2015). Asialoglycoprotein receptor mediated hepatocyte targeting- strategies and applications. J Controlled Release.

[39] Springer A D, Dowdy SF (2018). GalNAc-siRNA conjugates: leading the way for delivery of RNAi therapeutics. Nucleic Acid Ther.

[40] Sukumaran A, Lozovatsky L, Li X, Gonzalez LA, Cao C, Fleming MD (2017). NCOA4 acts in hepatocytes to regulate ferritin subunit levels and hepatic iron storage. Blood.

[41] Liu Y, Tan M, Fang C, Chen X, Liu H, Feng Y (2021). A novel multifunctional gold nanorod-mediated and tumor-targeted gene silencing of GPC-3 synergizes photothermal therapy for liver cancer. Nanotechnology.

[42] Xiao X, Wang Y, Chen J, Qin P, Chen P, Zhou D (2022). Self-targeting platinum (IV) amphiphilic prodrug nano-assembly as radiosensitizer for synergistic and safe chemoradiotherapy of hepatocellular carcinoma. Biomaterials.

[43] Marcocci ME, Napoletani G, Protto V, Kolesova O, Piacentini R, Li Puma DD (2020). Herpes simplex virus-1 in the brain: The dark side of a sneaky infection. Trends Microbiol.

[44] Gilliet M, Cao W, Liu YJ (2008). Plasmacytoid dendritic cells: sensing nucleic acids in viral infection and autoimmune diseases. Nat Rev Immunol.

[45] Mahmoud SMA, Paish EC, Powe DG, Macmillan RD, Grainge MJ, Lee AHS (2011). Tumor-infiltrating CD8+ lymphocytes predict clinical outcome in breast cancer. J Clin Oncol.

[46] Yoo JY, Jaime-Ramirez AC, Bolyard C, Dai H, Nallanagulagari T, Wojton J (2016). Bortezomib treatment sensitizes oncolytic HSV-1-treated tumors to NK cell immunotherapy oncolytic virus therapy in conjunction with bortezomib. Clin Cancer Res.

[47] Tanaka A, Sakaguchi S (2017). Regulatory T cells in cancer immunotherapy. Cell Res.

[48] Workenhe ST, Simmons G, Pol JG, Lichty BD, Halford WP, Mossman KL (2014). Immunogenic HSV-mediated oncolysis shapes the antitumor immune response and contributes to therapeutic efficacy. Mol Ther.

[49] Riera Romo M (2021). Cell death as part of innate immunity: Cause or consequence?. Immunology.

[50] Wakimoto H, Ikeda K, Abe T, Ichikawa T, Hochberg FH, Ezekowitz RAB (2002). The complement response against an oncolytic virus is species-specific in its activation pathways. Mol Ther.

[51] Nosaki K, Hamada K, Takashima Y, Sagara M, Matsumura Y, Miyamoto S (2016). A novel, polymer-coated oncolytic measles virus overcomes immune suppression and induces robust antitumor activity. Mol Ther Oncol.

[52] Xia M, Luo D, Dong J, Zheng M, Meng G, Wu J (2019). Graphene oxide arms oncolytic measles virus for improved effectiveness of cancer therapy. J Exp Clin Cancer Res.

[53] Walker S, Busatto S, Pham A, Tian M, Suh A, Carson K (2019). Extracellular vesicle-based drug delivery systems for cancer treatment. Theranostics.

[54] Kleemann J, Jäger M, Valesky E, Kippenberger S, Kaufmann R, Meissner M (2021). Real-world experience of talimogene laherparepvec (T-VEC) in old and oldest-old patients with melanoma: a retrospective single center study. Cancer Manag Res.

[55] Machiels JP, Salazar R, Rottey S, Duran I, Dirix L, Geboes K (2019). A phase 1 dose escalation study of the oncolytic adenovirus enadenotucirev, administered intravenously to patients with epithelial solid tumors (EVOLVE). J Immunother Cancer.

[56] Yu W, Geng S, Suo Y, Wei X, Cai Q, Wu B (2018). Critical role of regulatory T cells in the latency and stress-induced reactivation of HSV-1. Cell Rep.

[57] Chiocca EA, Rabkin SD (2014). Oncolytic viruses and their application to cancer immunotherapy. Cancer Immunol Res.

[58] Yan X, Zhang X, Wang Y, Li X, Wang S, Zhao B (2011). Regulatory T-cell depletion synergizes with gp96-mediated cellular responses and antitumor activity. Cancer Immunol Immunother.

[59] Yoo JY, Jaime-Ramirez AC, Bolyard C, Dai H, Nallanagulagari T, Wojton J (2016). Bortezomib treatment sensitizes oncolytic HSV-1-treated tumors to NK cell immunotherapy. Clin Cancer Res.

