# Supplementary material for: Systemic delivery of glycosylated-PEG-masked oncolytic virus enhances targeting of antitumor immuno-virotherapy and modulates T and NK cell infiltration
Source: Theranostics. 2023 Oct 2;13(15):5452–68. doi: 10.7150/thno.87498 (PMC10614686; doi:10.7150/thno.87498)
Supplement: Supplementary file 1 — Supplementary figures. [file thnov13p5452s1.pdf]

Supporting Information

Systemic delivery of glycosylated-PEG-masked oncolytic virus enhances targeting of antitumor immuno-virotherapy and modulates T and NK cell infiltration

Yuzhi Liang[1,5#], Bing Wang[1,5#], Qingjing Chen[1,4#], Xingyue Fu[1], Chenwei Jiang[1], Zhiwen Lin[1], Qiuyu Zhuang[1, 3], Yongyi Zeng[1,4], Xiaolong Liu[1,2,3*], Da Zhang[1, 3*]

[1]The United Innovation of Mengchao Hepatobiliary Technology Key Laboratory of Fujian Province, Mengchao Hepatobiliary Hospital of Fujian Medical University, Fuzhou 350025, P. R. China

[2]CAS Key Laboratory of Design and Assembly of Functional Nanostructures, Fujian Institute of Research on the Structure of Matter, Chinese Academy of Sciences, Fuzhou 350002, P. R. China

[3]Mengchao Med-X Center, Fuzhou University, Fuzhou 350116, P. R. China

[4]Liver Disease Center, The First Affiliated Hospital of Fujian Medical University, Fuzhou 350005, People's Republic of China

[5]Fujian Agriculture and Forestry University, Fuzhou 350002, P. R. China

[#]Y. L, B. W and Q. C contributed equally to this works

*Correspondence: Xiaolong Liu, Mengchao Hepatobiliary Hospital of Fujian Medical University, Fuzhou 350025, P. R. China

E-mail: xiaoloong.liu@gmail.com;

*Correspondence: Da Zhang, Mengchao Hepatobiliary Hospital of Fujian Medical University, Fuzhou 350025, P. R. China

E-mail: zdluoman1987@163.com;

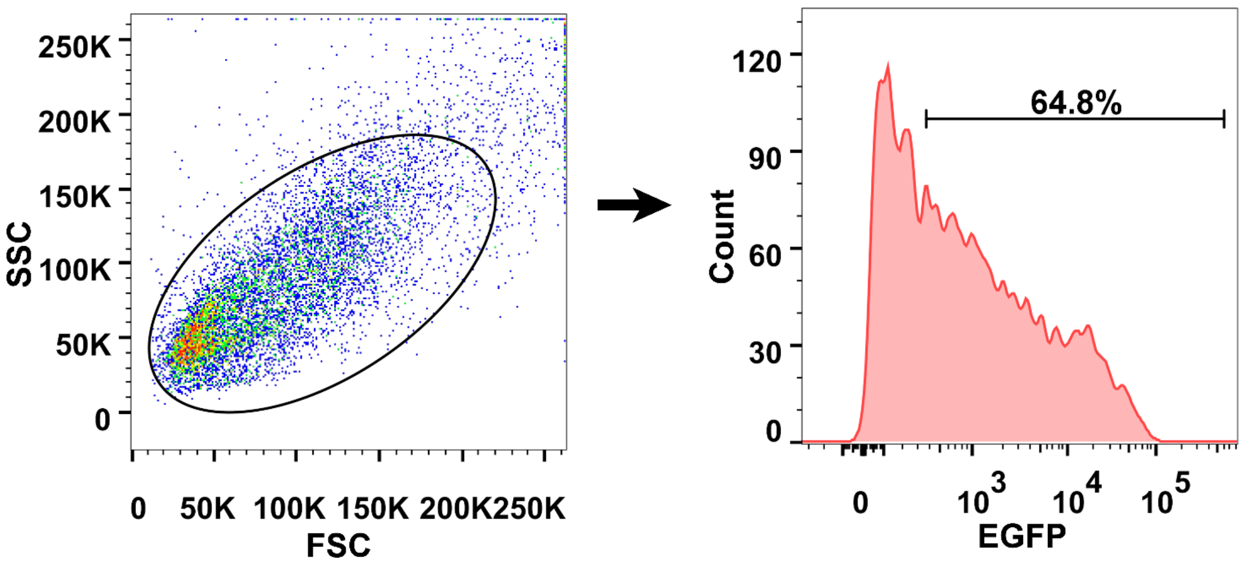

Figure. S1. The gate and EGFP intensity of oHSV-treated Hepa1-6 cells were analyzed by FACS.

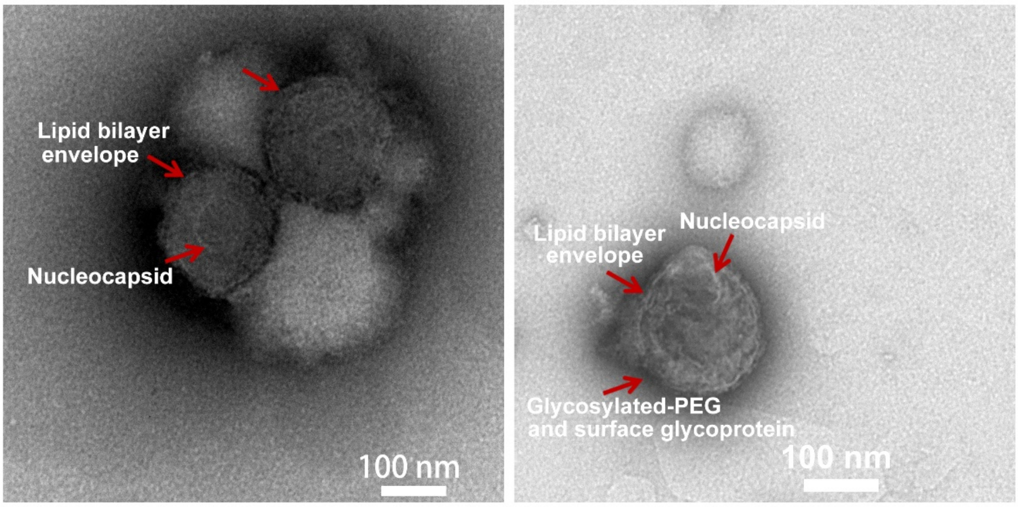

Figure S2. The TEM image of oHSV and glycosylated-PEG-oHSV.

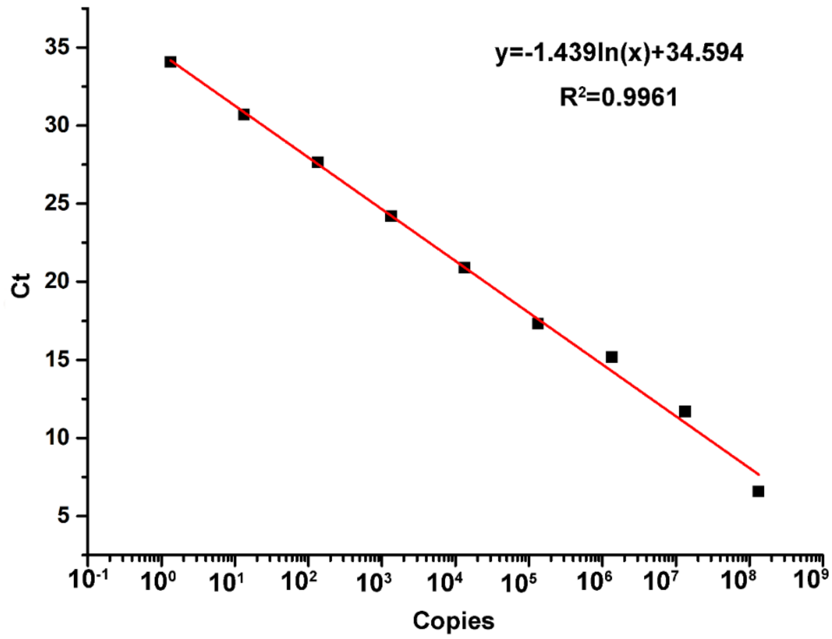

Figure. S3. Standard curve for virus copy number determination.

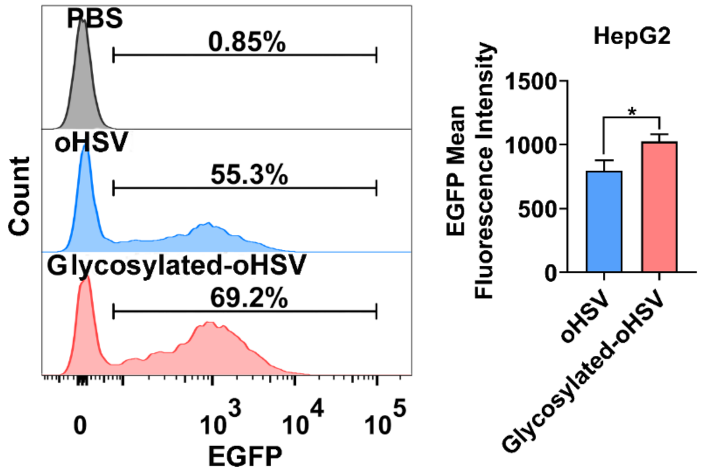

Figure S4. Flow cytometry was performed on HepG2 cells treated with oHSV or glycosylated-PEG-oHSV. Statistical analysis was performed with t-test, *$p<0.05$, (n = 3). Data are presented as mean ± SD.

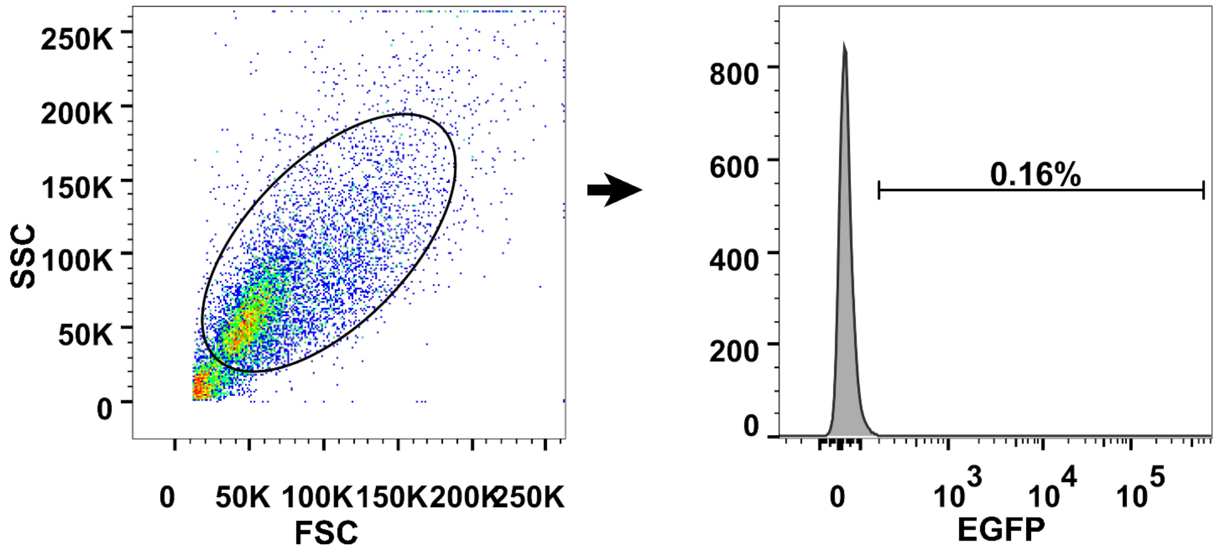

Figure. S5. The gate and EGFP intensity of NIH/3T3 cells treated with oHSV were analyzed by FACS.

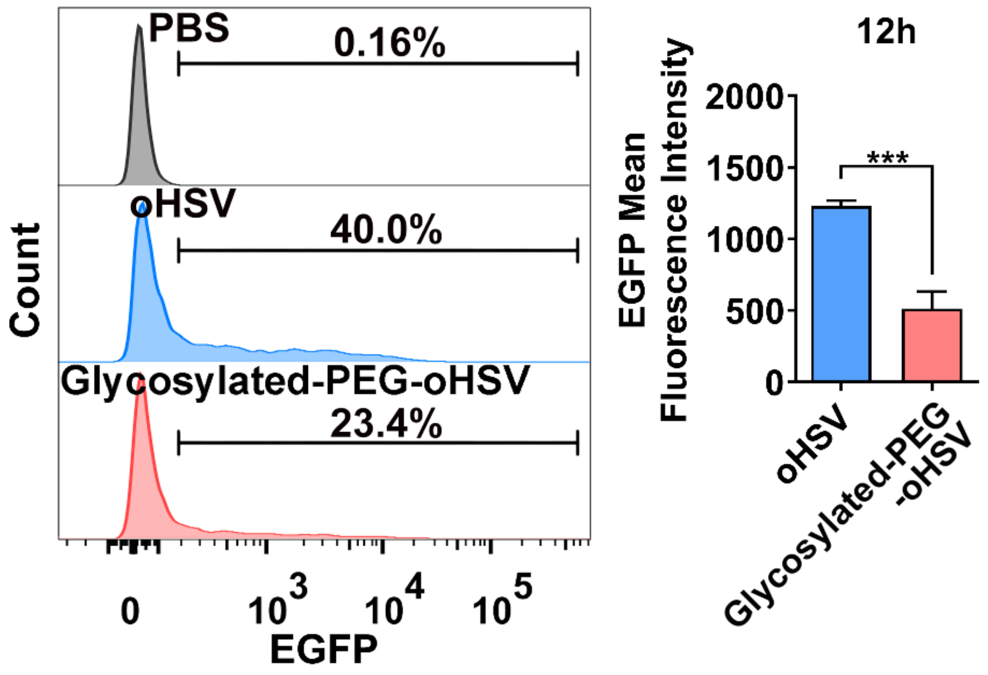

Figure. S6. Flow cytometry analysis and quantification of EGFP expression in NIH/3T3 cells after treatment with oHSV or glycosylated-PEG-oHSV for 12 h. Statistical analysis was performed using a t-test analysis, and significance levels were denoted by *$p<0.05$, **$p<0.01$, ***$p<0.001$, and ****$p<0.0001$. Data are presented as mean ± standard deviation (SD).

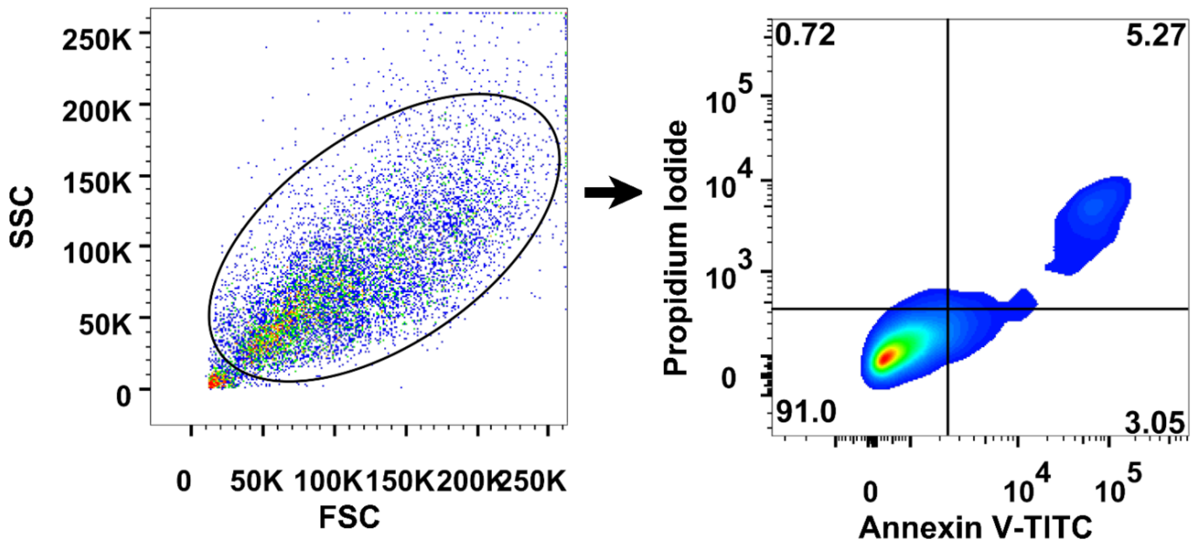

Figure. S7. The cytolytic activity of Hepa1-6 cells treated with oHSV for 24 h was analyzed by FACS using Annexin V-FITC and PI staining.

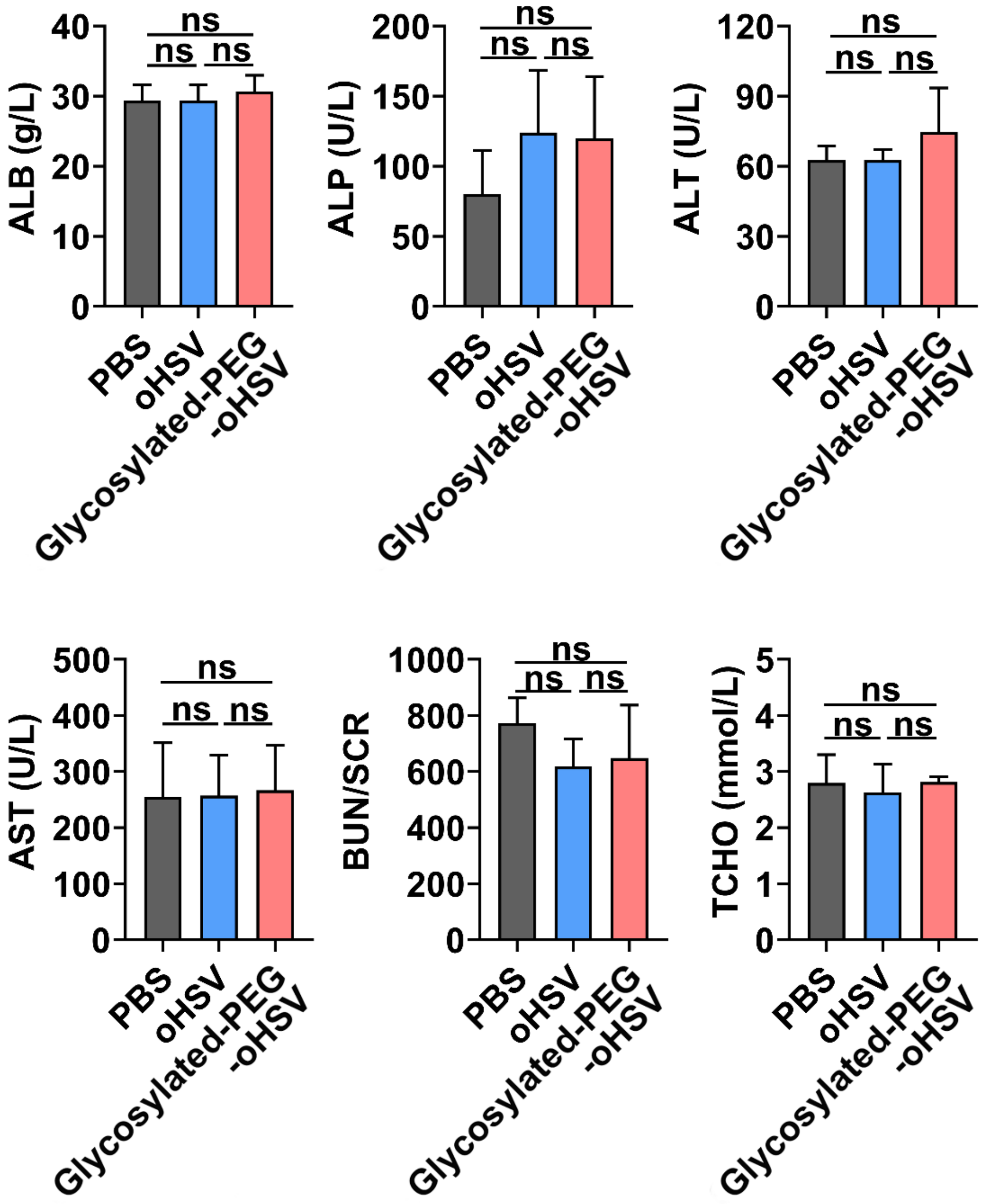

Figure. S8. Biochemical analysis, including albumin, ALB; alkaline phosphatase, ALP; alanine aminotransferase, ALT; aspartate aminotransferase, AST; the blood urea nitrogen to serum creatinine ratio (BUN/SCR); and total cholesterol (TCHO) was performed in mice at the day 24 after intravenous injection of PBS, oHSV, or glycosylated-PEG-oHSV (n=3). Statistical analysis was conducted using ANOVA analysis, with significance levels denoted as $*p<0.05$, $**p<0.01$, $***p<0.001$, *and* $****p<0.0001$. The data are presented as mean ± standard deviation (SD).

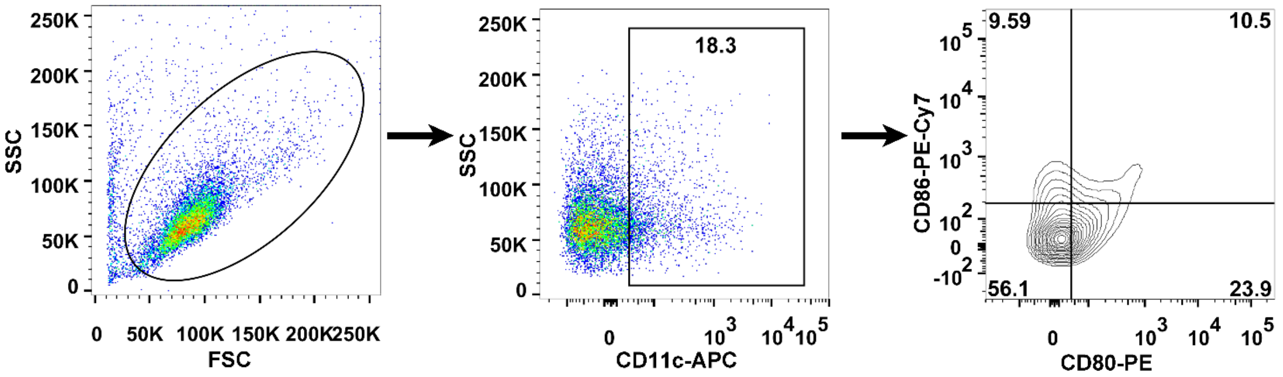

Figure. S9. Identifying the maturation of lymphatic dendritic cells (DCs) from lymph nodes of mice after receiving different treatments by FACS analysis with staining using anti-CD11c-APC, anti-CD80-PE, and anti-CD86-PE-Cy7 antibodies.

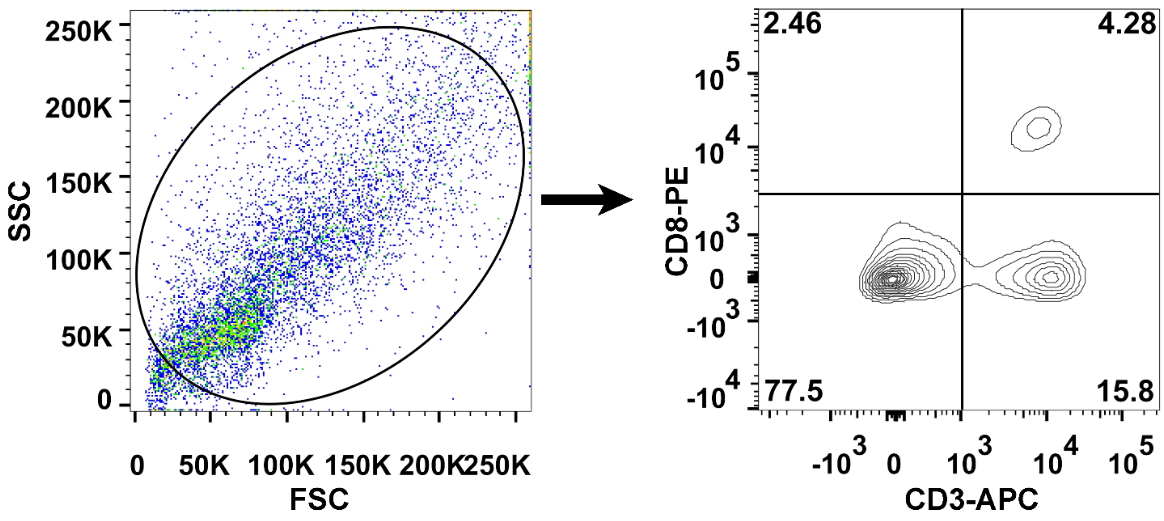

Figure. S10. Identifying the percentage of $CD8^{+}$T cells in spleen after receiving different treatments by FACS analysis with staining using anti-CD3-APC, anti-CD8-PE antibodies.

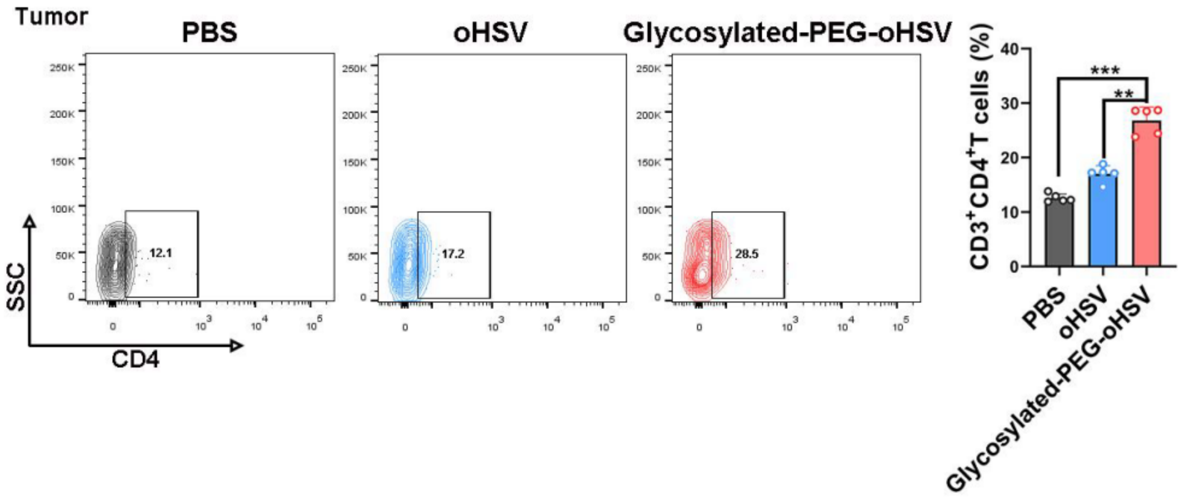

Figure S11. The percentage of CD3$^+$CD4$^+$T cells in tumors after treatment with PBS, oHSV or glycosylated- PEG-oHSV, respectively (n = 5).

Tumor

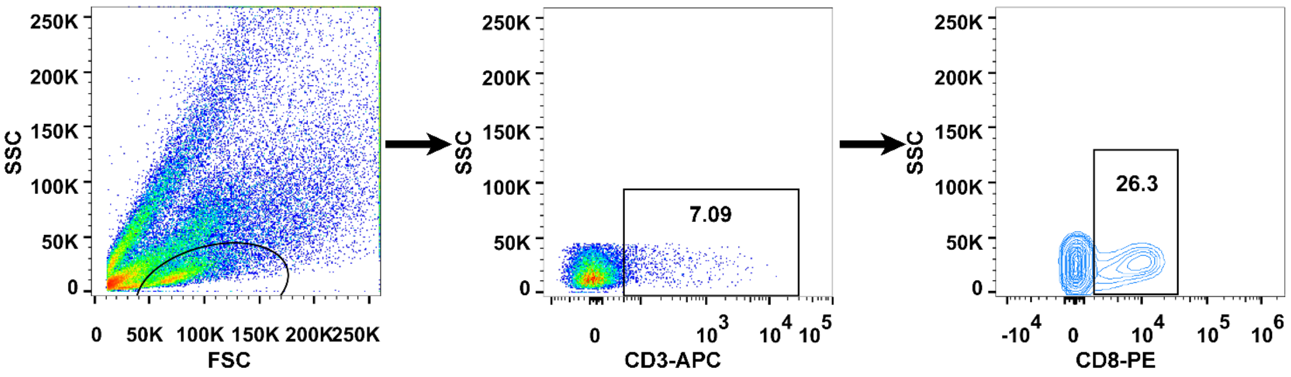

Figure. S12. Identifying the percentage of CD8$^+$T cells in tumors after receiving different treatments by FACS analysis with staining using anti-CD3-APC, anti-CD8-PE antibodies.

Tumor

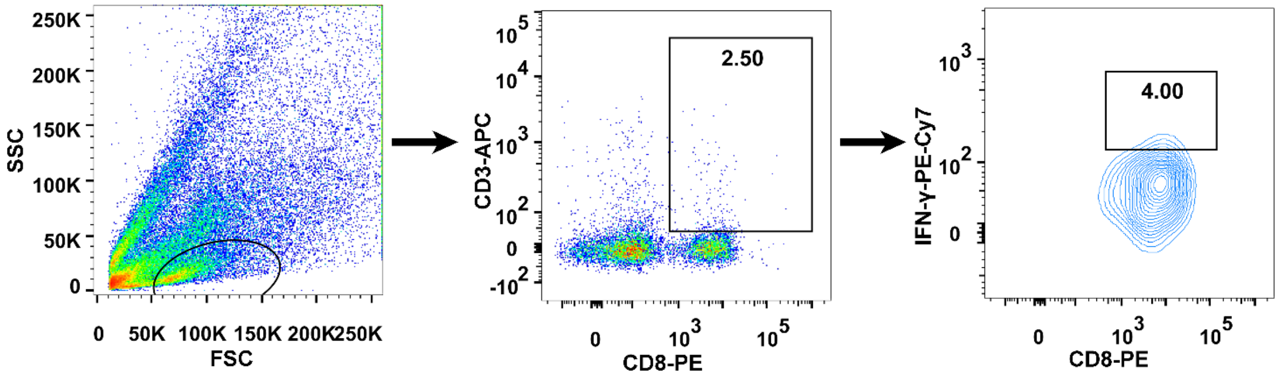

Figure. S13. Identifying IFN-γ+CD8+CD3+T cells in tumors after receiving different treatments by FACS analysis with staining using anti-CD3-APC, anti-CD8-PE, IFN-γ-PE-Cy7 antibodies.

Tumor

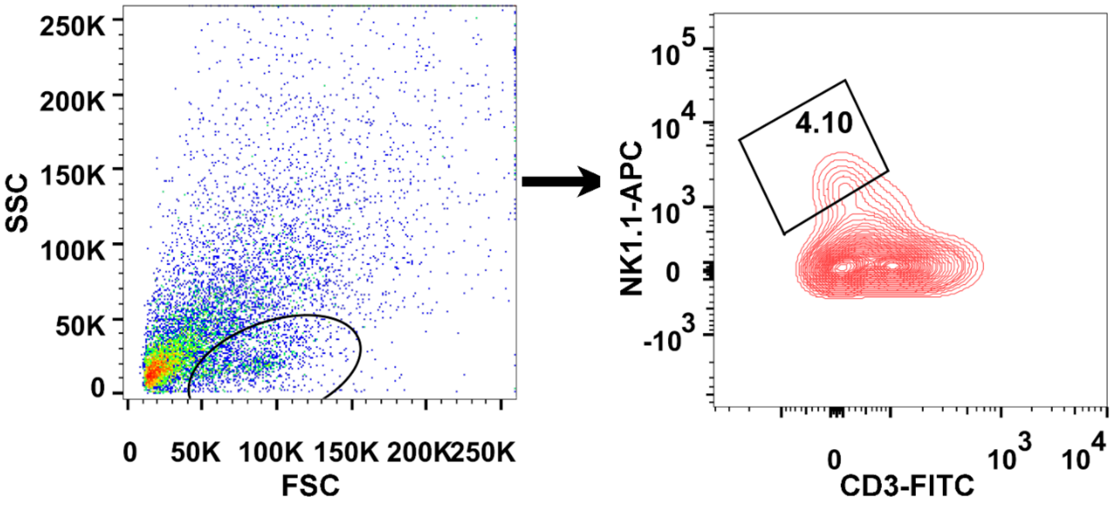

Figure. S14. Identifying NK cells in tumors after receiving different treatments by FACS analysis with staining using anti-CD3-FITC and anti-NK1.1-APC antibodies.

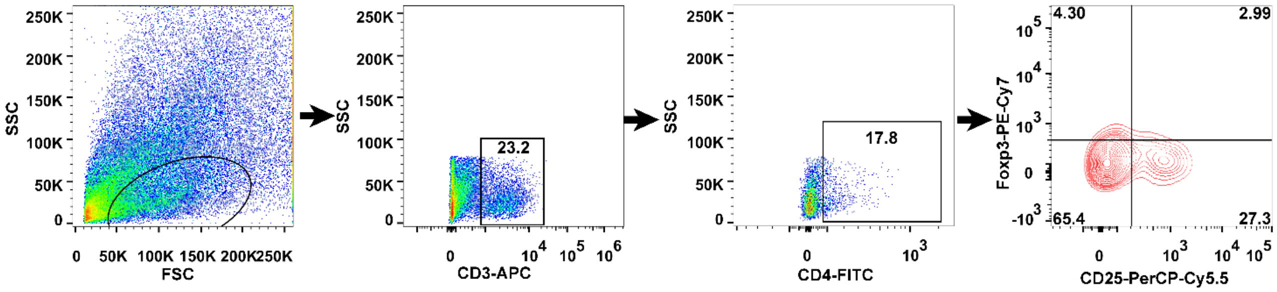

Figure. S15. Detection of Foxp3^{+}CD25^{+}CD4^{+}T cells in tumors following different treatments by FACS analysis with staining using anti-CD3-APC, anti-CD4-FITC, anti-CD25-PerCP-Cy5.5, and anti-Foxp3-PE-Cy7 antibodies.

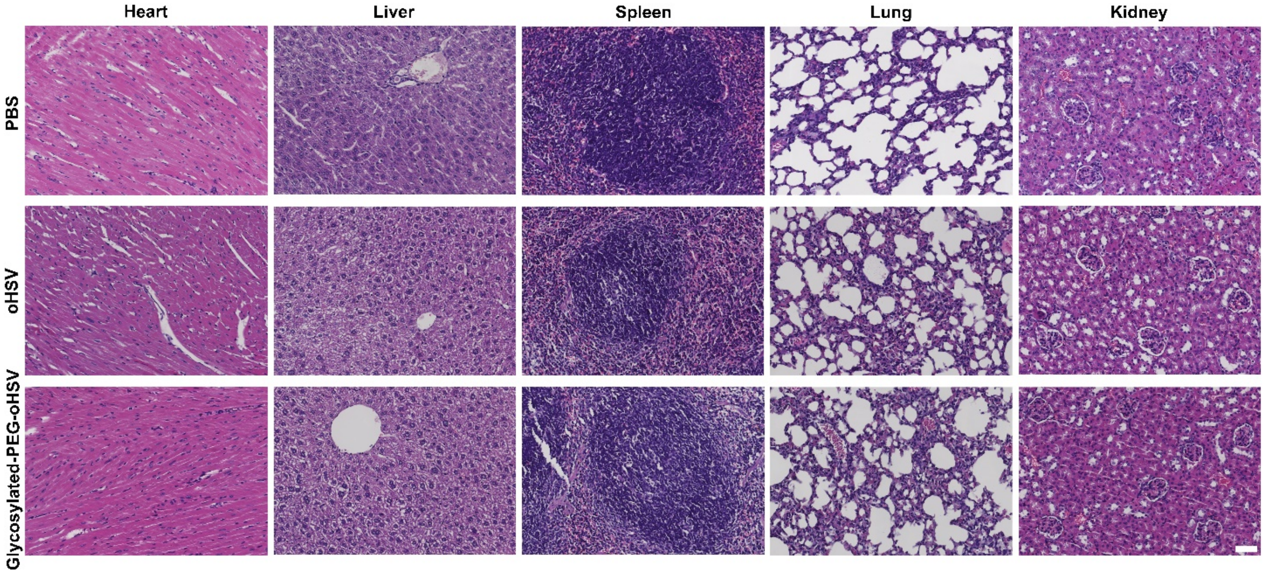

Figure. S16. Hematoxylin and eosin (H&E) imaging was performed on major organs including the heart, liver, spleen, lung, and kidney, obtained from mice at the day 24 after intravenous injection of PBS, oHSV, or glycosylated-PEG-oHSV. Scale bar, 100 μm.
